# *In vivo* functional analysis of a class A β-lactamase-related protein essential for clavulanic acid biosynthesis in *Streptomyces clavuligerus*

**DOI:** 10.1371/journal.pone.0215960

**Published:** 2019-04-23

**Authors:** Santosh K. Srivastava, Kelcey S. King, Nader F. AbuSara, Chelsea J. Malayny, Brandon M. Piercey, Jaime A. Wilson, Kapil Tahlan

**Affiliations:** Department of Biology, Memorial University of Newfoundland, St. John’s, NL, Canada; Rutgers New Jersey Medical School, UNITED STATES

## Abstract

In *Streptomyces clavuligerus*, the gene cluster involved in the biosynthesis of the clinically used β-lactamase inhibitor clavulanic acid contains a gene (*orf12* or *cpe*) encoding a protein with a C-terminal class A β-lactamase-like domain. The *cpe* gene is essential for clavulanic acid production, and the recent crystal structure of its product (Cpe) was shown to also contain an N-terminal isomerase/cyclase-like domain, but the function of the protein remains unknown. In the current study, we show that Cpe is a cytoplasmic protein and that both its N- and C-terminal domains are required for *in vivo* clavulanic acid production in *S*. *clavuligerus*. Our results along with those from previous studies allude towards a biosynthetic role for Cpe during the later stages of clavulanic acid production in *S*. *clavuligerus*. Amino acids from Cpe essential for biosynthesis were also identified, including one (Lys_89_) from the recently described N-terminal isomerase-like domain of unknown function. Homologues of Cpe from other clavulanic acid-producing *Streptomyces* spp. were shown to be functionally equivalent to the *S*. *clavuligerus* protein, whereas those from non-producers containing clavulanic acid-like gene clusters were not. The suggested *in vivo* involvement of an isomerase-like domain recruited by an ancestral β-lactamase related protein, supports a previous hypothesis that Cpe could be involved in a step requiring the opening and modification of the clavulanic acid core during its biosynthesis from 5*S* precursors.

## Introduction

**Fig 1:**
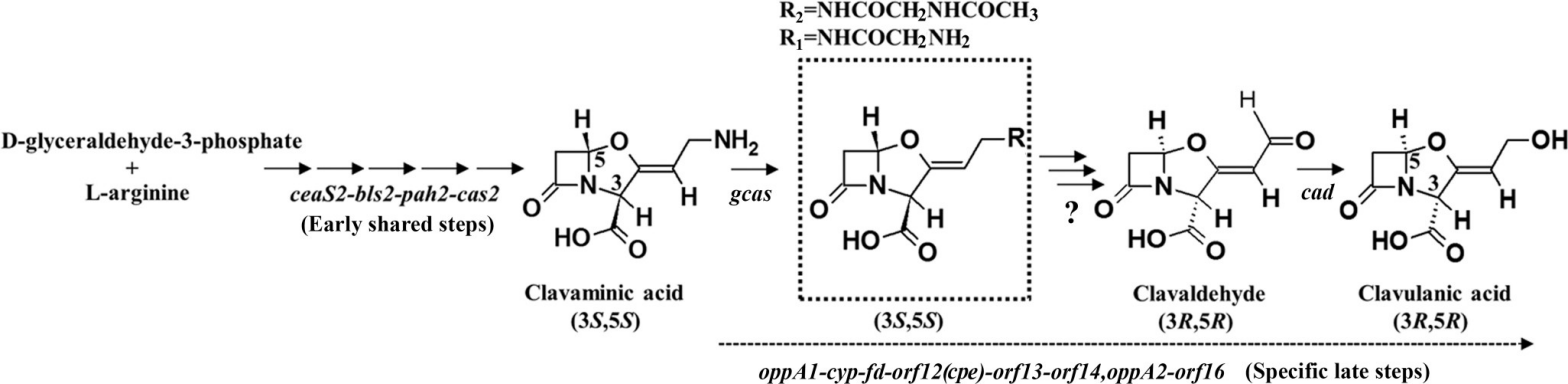
Diagrammatic representation of the partial clavulanic acid biosynthetic pathway from *Streptomyces clavuligerus*. Genes encoding enzymes known to be involved in the “early” shared stages of 5*S* clavam and clavulanic acid production, and those predicted to encode proteins involved exclusively in the biosynthesis of clavulanic acid (“late” steps) are indicated. In addition, genes encoding enzymes with known biosynthetic functions are shown next to arrows representing the respective reactions catalyzed by them, and the question mark indicates the unknown protein(s) responsible for the 5*S* to 5*R* epimerization and side chain modification of clavam intermediates during clavulanic acid biosynthesis. The two 5*S* clavam intermediates related to clavaminic acid (R_1_ = *N*-glycyl and R_2_ = *N*-acetyl-glycyl, respectively), which accumulate in the *orf15* and *orf16* gene mutants are shown in the dashed box.

The β-lactam class of antibiotics have broad-spectrum activity and include some of the most commonly prescribed agents used for treating bacterial infections [1–3]. They have a long history of use in medicine, but as with other antibiotics, the emergence of resistance is a major problem [3–5]. There are several mechanisms responsible for β-lactam resistance, which include the production of secreted β-lactamases, enzymes that hydrolyze and inactivate certain members of this antibiotic class [6, 7]. Combinations of β-lactamase inhibitors such as clavulanic acid along with β-lactam antibiotics are often used as a strategy for treating some infections caused by β-lactamase-producing antibiotic resistant bacteria [8, 9]. Clavulanic acid belongs to the clavam family of specialized metabolites and it irreversibly inhibits class A β-lactamases, thereby restoring the activity of β-lactam antibiotics against target organisms in such combinations [10, 11]. The activity of clavulanic acid is attributed in part to its 3*R*,5*R* stereochemistry, as other naturally occurring clavams have a 5*S* configuration (collectively referred to as the 5*S* clavams) and do not inhibit β-lactamases [8, 12]. Commercial production of clavulanic acid is achieved by fermenting *Streptomyces clavuligerus*, and a cluster of ∼18 genes referred to as the clavulanic acid biosynthetic gene cluster (CA-BGC) encodes components of the core biosynthetic pathway [13]. It has previously been reported that *Streptomyces jumonjinensis* and *Streptomyces katsurahamanus* also produce clavulanic acid, but the sequences of their respective CA-BGCs are not available [12, 14]. On the other hand, the genome sequences of organisms such as *Streptomyces flavogriseus* (ATCC 33331, also known as *S*. *pratensis*) and *Saccharomonospora viridis* (DSM 43017) contain gene clusters closely resembling the *S*. *clavuligerus* CA-BGC, but neither has been shown to produce the metabolite to date [13, 15]. In addition, *S*. *clavuligerus* is somewhat unique among clavulanic acid producers as it also produces certain 5*S* clavams as products of a pathway related to clavulanic acid [13, 16]. Clavulanic acid and the 5*S* clavams have common biosynthetic origins and the pathway involved in their production can be roughly divided into two parts in *S*. *clavuligerus* (Fig 1). The “early” steps leading up to the intermediate clavaminic acid are shared during the production of both types of metabolites, with all intermediates possessing 5*S* configuration [17]. Beyond clavaminic acid (also a 5*S* clavam) the pathway diverges into specific “late” steps leading to either the 5*S* clavams or to clavulanic acid (Fig 1) [18].

The early shared portion of the pathway has been well characterized along with the genes involved in the process [19], but specific reactions involved in the production of each type of metabolite are yet to be elucidated [13]. It is currently hypothesized that during clavulanic acid production, the intermediate clavaminic acid undergoes oxidative deamination and ring inversion leading to clavaldehyde (Fig 1), which has 5*R* stereochemistry and is the immediate precursor of clavulanic acid [20]. The enzymes responsible for clavaldehyde formation are not known, but the products of *orf10-17* from the CA-BGC are thought to play a role in the process [13, 17]. Previous reports have shown that the disruption of individual genes from the *orf10-17* region abolishes or reduces clavulanic acid production without affecting 5*S* clavam levels [21–23]. Under certain conditions, the concomitant accumulation of acylated clavaminic acid derivatives was also observed in the *orf15*-*16* mutants [23, 24], suggesting that the respective metabolites are intermediates from the clavulanic acid arm of the biosynthetic pathway (Fig 1). Because of the clinical applications of clavulanic acid, there is considerable interest in understanding how the metabolite is produced in *S*. *clavuligerus*.

**Fig 2:**
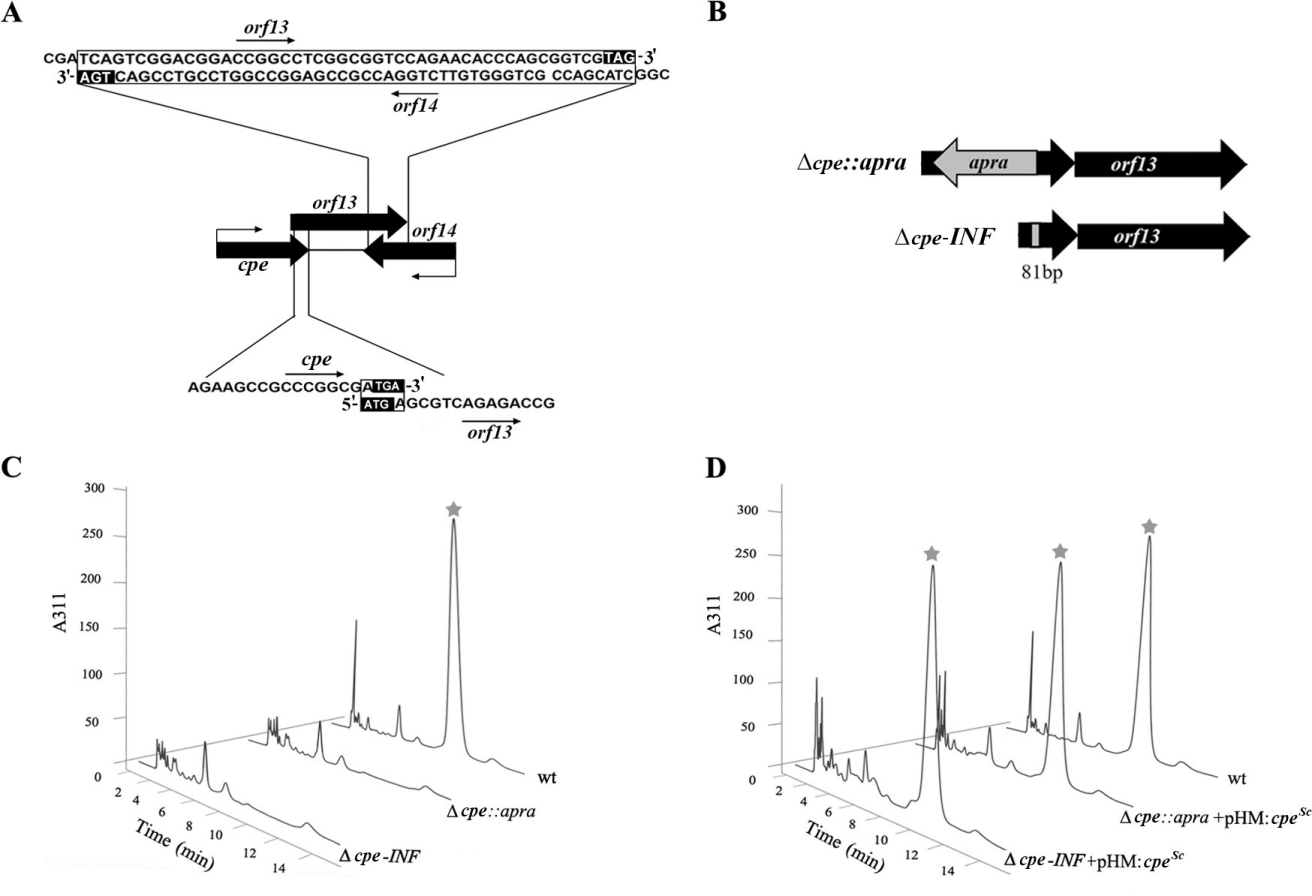
Preparation and analysis of *S*. *clavuligerus cpe* deletion mutants. (A and B) The thick arrows depict genes with arrowheads indicating the direction of transcription. (A) The relative arrangement of genes from the chromosomal locus surrounding *cpe* in *S*. *clavuligerus* is shown, and the bent arrows represent the known promoters for the different transcriptional units. The DNA sequences of the overlapping regions between *cpe*-*orf13* (bottom) and *orf13*-*orf14* (top) are indicated in open boxes, whereas the respective start and stop codons are shown in filled boxes. (B) Diagrammatic representation of the *Δcpe*::*apra* and *Δcpe-INF* mutants, which were prepared such that the 5' and 3' ends of *cpe* were retained and intervening DNA sequences were replaced by an apramycin resistance cassette or an in-frame 81-bp sequence in the respective mutants. (C and D) HPLC analysis of 96 hour wt *S*. *clavuligerus* and different *cpe* mutant SA culture supernatants for assessing clavulanic acid production using the phosphate buffer system [32]. Peaks corresponding to imidazole-derivatized clavulanic acid are indicated by the star symbol, which were detected at 311nm.

Of particular relevance to the current study is the product of *orf12* (SCLAV_4187) from the CA-BGC of *S*. *clavuligerus*, which resembles class A β-lactamases and also contains similar SXXK, SDN and KAG amino acid motifs [25]. *orf12* is co-transcribed with *orf13*, which encodes a putative membrane transport protein (Fig 2A), and their relative arrangement also suggests possible translational coupling [23]. Due to the bioactivities of specialized metabolites (especially when the product is an antibacterial), producer organisms often employ self-resistance strategies for protection [26, 27]. Intrinsic resistance in β-lactam-producing organisms is often attributed to the presence of altered penicillin-binding proteins (PBPs, the targets of β-lactam antibiotics) with reduced binding affinities for endogenously-produced antibiotics [28], but BGCs from such organisms also contain genes encoding β-lactamases and efflux transporters [29, 30]. Studies have shown that *orf12* is required for clavulanic acid, but not 5*S* clavam production [23] and that the encoded protein lacks any detectible β-lactamase activity. Instead, heterologously expressed and purified Orf12 was shown to function as a cephalosporin esterase under *in vitro* conditions [25], due to which it is henceforth referred to as Cpe (for cephalosporin esterase).

The recently solved crystal structure of Cpe showed that in addition to a β-lactamase-like domain located in its C-terminus (residues 128–458), the protein also contains a previously unrecognized N-terminal domain (residues 1–127) resembling those found in steroid isomerases and polyketide cyclases [25]. In addition, two molecules of clavulanic acid were found to be bound non-covalently to Cpe when crystals of the protein were soaked in a solution of the metabolite during structural studies [25]. The first molecule (CA-1) was positioned in an active site pocket lined by residues (His88, Ser173, Thr209, Ser234, Ser278,Met383, Phe374, Ala376 and Phe385) from both the N- and the C-terminal domains, whereas the second molecule (CA-2) bound to a mostly hydrophobic cleft at the interface of the two domains *via* weak ionic interactions [25]. It was also shown that apart from Ser_173_, Ser_234_ and Ser_378_, other residues from Cpe or its N-terminal domain are not essential for its *in vitro* esterase activity. The ability of Cpe to bind clavulanic acid non-covalently under *in vitro* conditions is intriguing [25], as bona fide class A β-lactamases form irreversible covalent suicide adducts with the inhibitor [9]. In addition, β-lactamases are secreted out of the cell to inactive their antibiotic substrates [7], but the cellular location of Cpe in *S*. *clavuligerus* is not known. It is also not clear if Cpe undergoes post-translational processing in *S*. *clavuligerus*, or if both of its N- and C-terminal domains and associated amino acid residues are required for clavulanic acid production in the native host. Therefore, questions regarding the actual *in vivo* role of the Cpe gene product still remain unanswered, many of which are examined in the current study.

## Materials and methods

### Bacterial strains, plasmids and culture conditions

Dehydrated media components and reagents were purchased from VWR International, Fisher Scientific or Sigma-Aldrich (Canada). Details of bacterial strains and plasmids used in the current study are described in Tables 1 and 2, respectively. *Escherichia coli* and *S*. *clavuligerus* cultures were grown and manipulated as described previously [31, 32]. Other *Streptomyces* species were cultured using tryptic soy broth (TSB) or ISP4 media, whereas *S*. *viridis* was grown in nutrient broth (BD, Canada). Unless otherwise specified, all *E*. *coli*, *Streptomyces* and *S*. *viridis* cultures were grown at 37, 28 and 42°C, respectively. Appropriate antibiotics were included in the media when required [31, 33], and liquid cultures were agitated at 200 rpm. For assessment of metabolite production, *S*. *clavuligerus* strains were grown in duplicate in starch asparagine (SA) or soy fermentation media as described previously [19]. All production phenotypes were verified using at least two independent fermentations.

**Table 1:** Bacterial strains used in the current study.

Bacterial strain	Antibiotic resistance marker(s)^a^	Description	Source/Reference^b^
***Escherichia coli* strains**			
*E*. *coli* NEB5α	NA	DH5α derived cloning host	NEB
*E*. *coli* BL21(DE3)	NA	Host for protein expression	NEB
*E*. *coli* ET12567(pUZ8002)	Cam^R^, Kan^R^	DNA methylation deficient conjugation host containing the plasmid pUZ8002	[33]
*E*. *coli* BW25118 (pIJ790)	Cam^R^, Kan^R^	Host containing the plasmid pIJ790 for λ RED mediated ReDirect PCR targeting of genes	[34]
*E*. *coli* DH5α (BT340)	Amp^R^, Cam^R^	Strain containing plasmid BT340 used for expressing the FLP recombinase	[35]
***Streptomyces* and other strains**			
*Streptomyces clavuligerus* NRRL 3585	NA	Wild type; cephamycin and clavulanic acid producer	NRRL
*Streptomyces clavuligerus Δcpe*::*apra*	Apr^R^	*cpe* deletion mutant; gene replaced by disruption cassette from plasmid pIJ773	This study
*Streptomyces clavuligerus Δcpe*-*INF*	NA	*cpe* deletion mutant; gene replaced by 81bp marker less in-frame scar sequence	This study
*Streptomyces flavogriseus* ATCC 33331	NA	Wild type; clavulanic acid non-producer	ATCC
*Saccharomonospora viridis* ATCC 15386	NA	Wild type; clavulanic acid non-producer	ATCC
*Streptomyces katsurahamanus*	NA	Wild type; cephamycin and clavulanic acid producer	[12]
*Streptomyces jumonjinensis*	NA	Wild type; cephamycin and clavulanic acid producer	[12]
*Klebsiella pneumoniae* ATCC 15380	NA	Indicator organism for clavulanic acid bioassays	[32]

^a^ Amp^R^, ampicillin resistance; Apr^R^, apramycin resistance; Cam^R^, chloramphenicol resistance; Kan^R^, kanamycin resistance; NA, Not applicable.

^b^ ATCC, American Type Culture Collection; NEB, New England Biolabs; NRRL, Northern Regional Research Laboratory.

**Table 2:** Plasmids and cosmids used in the current study.

Plasmid/cosmid	Antibiotic resistance marker(s)^a^	Description	Source/Reference
pGEMT-Easy	Amp^R^	General *E*. *coli* cloning vector	Promega
pET30b	Kan^R^	*E*. *coli* protein expression vector	Novagen
pHM11a	Hyg^R^	Integrative *Streptomyces* expression vector containing the constitutive *ermE*p*	[36]
pSET152	Apr^R^	Integrative *Streptomyces* cloning vector	[37]
pIJ773	Apr^R^	Template plasmid for preparing the ReDirect *apra* disruption cassette	[34]
pIJ10700	Hyg^R^	Template plasmid for preparing the ReDirect *hyg* disruption cassette	[34]
pET30b-*cpe*^*Sc*^	Kan^R^	Plasmid vector used to express C-terminal 6×His-tagged Cpe in *E*. *coli* for purification	This study
12B8	Amp^R^, Kan^R^	Cosmid clone containing the clavulanic acid biosynthetic gene cluster from *S*. *clavuligerus*	[19]
12B8-*Δcpe*::*apra*	Apr^R^, Amp^R^, Kan^R^	Mutant cosmid 12B8 in which *cpe* has been replaced by the disruption cassette from plasmid pIJ773 using the ReDirect system	This study
12B8-*Δcpe-INF*	Amp^R^, Kan^R^	Mutant cosmid 12B8 in which *cpe* has been replaced by the 81-bp in-frame “scar” sequence using the ReDirect system	This study
12B8-*Δcpe-INF-Δamp*::*hyg*	Hyg^R^, Kan^R^	Cosmid 12B8-*Δcpe-INF* in which ampicillin resistance gene replaced by the *hyg* cassette from plasmid pIJ10700 using the ReDirect system	This study
pHM:*cpe*^*Sc*^	Hyg^R^	Expression plasmid pHM11a containing *cpe* from *S*. *clavuligerus*	This study
pHM:*cpe*^*Sf*^	Hyg^R^	Expression plasmid pHM11a containing *cpe* from *S*. *flavogriseus*	This study
pHM:*cpe*^*Sv*^	Hyg^R^	Expression plasmid pHM11a containing *cpe* from *S*. *viridis*	This study
pHM:*cpe*^*Sj*^	Hyg^R^	Expression plasmid pHM11a containing *cpe* from *S*. *jumonjinensis*	This study
pHM:*cpe*^*Sk*^	Hyg^R^	Expression plasmid pHM11a containing *cpe* from *S*. *katsurahamanus*	This study
pHM:*blip*^*FLAG*^	Hyg^R^	Expression plasmid pHM11a containing *blip* from *S*. *clavuligerus* with a C-terminal FLAG tag	This study
pHM:*ccaR*^*FLAG*^	Hyg^R^	Expression plasmid pHM11a containing *ccaR* from *S*. *clavuligerus* with a C-terminal FLAG tag	This study
pHM:*cpe*^*Sc-FLAG*^	Hyg^R^	Expression plasmid pHM11a containing *cpe* from *S*. *clavuligerus* with a C-terminal FLAG tag	This study
pHM:*cpe*^*Sc-6×his*^	Hyg^R^	Expression plasmid pHM11a containing *cpe* from *S*. *clavuligerus* with a C-terminal 6×His tag	This study
pHM:*cpe*^*Ct*^	Hyg^R^	Expression plasmid pHM11a containing the C-terminal domain of *cpe* from *S*. *clavuligerus*	This study
pHM:*cpe*^*Nt*^	Hyg^R^	Expression plasmid pHM11a containing the N-terminal domain of *cpe* from *S*. *clavuligerus*	This study
pHM*-cpe*^*Ct+Nt*^	Hyg^R^	Expression plasmid pHM11a containing the N-terminal and C- terminal domains of *cpe* from *S*. *clavuligerus*, each expressed independently under the control of the *ermE*p*	This study
pSET:*cpe*^*Sc*^	Apr^R^	Plasmid pSET152 containing the *S*. *clavuligerus cpe* gene along with *ermE*p* from pHM11a was used as template to prepare all described *cpe*^*Sc*^ site directed mutants	This study

^a^Amp^R^, ampicillin resistance; Apr^R^, apramycin resistance; Kan^R^, kanamycin resistance, Hyg^R^, hygromycin resistance.

### DNA isolation, manipulation and analysis

All oligonucleotide primers used in the current study were purchased from Integrated DNA Technologies (USA) and are listed in S1, S2 and S3 Tables. Standard techniques were used to introduce, isolate, manipulate and analyze plasmid DNA from *E*. *coli* (35). Restriction enzymes used in the study were purchased from New England Biolabs Ltd. (Canada). Chromosomal DNA was isolated from *Streptomyces* and *S*. *viridis* cultures using the QIAamp DNA Mini Kit (QIAGEN, Canada) and a SpeedMill PLUS Bead Homogenizer (Analytik Jena, Germany), which was also used in all subsequent bead-beating purposes. PCR was performed using either the Fisher BioReagents *Taq* DNA polymerase or the Phusion High-Fidelity DNA Polymerase kits (Fisher Scientific, Canada) according to the manufacturer’s recommendations, except that 5% DMSO was included in problematic reactions. DNA fragments were purified after standard TBE agarose gel electrophoresis using the EZ-10 Spin Column DNA Gel Extraction Kit according to the manufacturer’s instructions (Bio Basic Canada Inc.). Unless otherwise specified, all PCR products were cloned into the pGEM-T Easy vector (Promega, USA) and the DNA sequences of all inserts were determined at the Centre for Applied Genomics, University of Toronto, Canada. Plasmid and cosmid constructs were introduced into *S*. *clavuligerus* through intergeneric conjugation using *E*. *coli* ET12567/pUZ8002 as described previously [19, 33].

### Preparation of the *S*. *clavuligerus Δcpe*::*apra* and *Δcpe-INF* mutants

The pWE15 vector based cosmid clone 12B8 (Table 2) containing the entire clavulanic acid gene cluster was used to prepare the *S*. *clavuligerus Δcpe* mutants according to the previously described ReDirect PCR-Targeting method [19, 34]. Specific oligonucleotide primers (S1 Table) along with pIJ773 as template were used to amplify a PCR product containing the apramycin resistance cassette (*apra*) to target *cpe* in 12B8. This led to the replacement of an internal fragment of *cpe* by the *apra* disruption cassette to give the mutant cosmid 12B8-*Δcpe*::*apra*. In addition, the *apra* cassette comprising the *aac3(IV)* gene and RK2 *oriT* flanked by FLP recombinase target sites (FRT), was inserted in the direction opposite to *cpe* transcription in the mutant cosmid. 12B8-*Δcpe*::*apra* was then introduced into wt *S*. *clavuligerus* for double homologous recombination and isolation of the apramycin resistant, *Δcpe*::*apra* mutant.

In order to prepare the in-frame (*INF*) marker-less *Δcpe-INF* mutant, cosmid 12B8-*Δcpe*::*apra* from above was introduced in *E*. *coli* DH5α/BT340, which expresses the FLP recombinase [35]. FLP caused the excision of the FRT-flanked *apra* cassette in 12B8-*Δcpe*::*apra*, leaving an 81-bp in-frame DNA sequence (“scar”) in its place in the mutant cosmid 12B8-*Δcpe-INF* (Table 2). Since *oriT* is part of the *apra* cassette, it was also lost, and 12B8-*Δcpe-INF* could not be transferred to *S*. *clavuligerus via* conjugation. Therefore, an *oriT* was introduced into 12B8-*Δcpe-INF* using a second round of ReDirect PCR-Targeting [34]. Specified primers (S1 Table) were used along with pIJ10700 as a template to amplify a PCR product containing the hygromycin resistance cassette (*hyg*) to target the ampicillin resistance gene present on the pWE15 vector backbone of 12B8-*Δcpe-INF*. The resulting cosmid 12B8-*Δcpe-INF-Δamp*::*hyg* (Table 2), containing the *hyg* cassette (which in turn contains an *oriT*) in place of the ampicillin resistance gene, was transferred to the *S*. *clavuligerus Δcpe*::*apra* mutant by conjugation with *E*. *coli*. Hygromycin-resistant colonies that arose were then made to undergo sporulation without any antibiotic selection to isolate the apramycin and hygromycin sensitive *S*. *clavuligerus Δcpe-INF* mutant. The replacement of the wt *cpe* gene with *Δcpe*::*apra* and *Δcpe-INF* in the respective *S*. *clavuligerus* mutants was confirmed by genomic DNA PCR and sequencing of products using specific primers (S1 Table).

### Preparation of *cpe* complementation plasmids

Specific oligonucleotide primers (S1 Table) with engineered NdeI and HindIII/BamHI restriction sites were used to PCR amplify DNA fragments containing the *cpe* genes from *S*. *clavuligerus* (^*Sc*^), *S*. *jumonjinensis* (^*Sj*^), *S*. *katsurahamanus* (^*Sk*^), *S*. *flavogriseus* (^*Sf*^) and *S*. *viridis* (^*Sv*^) for complementation studies. Since the sequences of *cpe* from *S*. *jumonjinensis* and *S*. *katsurahamanus* were not know, degenerate oligonucleotide primers with engineered restriction sites were designed based on known *cpe* DNA sequences from the three other species. After PCR amplification, the DNA fragments were directly cloned into the NdeI and HindIII/BamHI sites of the *Streptomyces* expression plasmid pHM11a [36] to give pHM:*cpe*^*Sj*^, pHM:*cpe*^*Sk*^, pHM:*cpe*^*Sf*^ and pHM:*cpe*^*Sv*^ (Table 2). The DNA sequences of all inserts were also verified/determined for comparison using custom primers (S1 Table).

To examine the *in vivo* roles of the N- and C-terminal domains of Cpe^Sc^, custom oligonucleotide primers were used to amplify DNA fragments containing each domain separately (S1 Table). The respective PCR fragments were cloned into pHM11a at NdeI and BamHI after their sequences had been verified to give pHM:*cpe*^*Nt*^ and pHM:*cpe*^*Ct*^, which functioned as the Cpe^*Sc*^ N- and C-terminal domain expression constructs, respectively (Table 2). To prepare a construct that could express the two domains separately at the same time from a single plasmid, the insert from pHM:*cpe*^*Ct*^ was released as a BglII-BamHI fragment and ligated to BamHI-digested pHM:*cpe*^*Nt*^. This led to the plasmid pHM:*cpe*^*Nt+*Ct^, in which the expression of each domain (not as part of the same protein) was driven independently by *ermE*p* (Table 2). Plasmid constructs were introduced into either the *S*. *clavuligerus Δcpe*::*apra* and/or *Δcpe-INF* mutants for complementation studies.

### Detection and localization of Cpe^Sc^ in *S*. *clavuligerus*

Engineered oligonucleotide primers were used to add C-terminal FLAG tags onto Cpe, CcaR and Blip (S1 Table). PCR fragments containing the three respective genes (*cpe*^*Sc-FLAG*^, *ccaR*^*FLAG*^ and *blip*^*FLAG*^) were cloned into pHM11a and introduced into wt *S*. *clavuligerus* for localization studies (Table 2). One hundred milliliters *S*. *clavuligerus* SA cultures expressing each protein were separately grown for 48 hours, after which the cultures were subjected to centrifugation and the mycelial pellets were separated from the supernatants. Cell pellets were resuspended in 5 ml of lysis buffer (150 mM HEPES and 150 mM NaCl) and were sonicated on ice using a 5/64-inch probe (VWR International, Canada). The lysates were centrifuged at high-speed (27,000 × *g*) for 15 minutes to clarify the cytoplasmic fraction contained in the supernatants for subsequent use. Approximately 87 ml of culture supernatant (separated from the above mycelial pellet in the first step) was centrifuged at 27,000 × *g* for 15 minutes and was then filtered through 0.2 μm vacuum membranes (VWR International, Canada) to remove any residual particulate or insoluble material. To precipitate secreted proteins, 44.9 g of ammonium sulfate was added gradually to 500 ml of the filtered supernatant (final volume is made up by using lysis buffer) with constant stirring at 4°C to give 80% saturation. Precipitated protein fractions were collected by high-speed centrifugation as described above, after which the supernatant was discarded, and the protein pellet was left to air dry for 10 minutes. The pellet was then resuspended in 500 μl of 1M phosphate buffer (sodium phosphate, pH-7.0) for future analysis.

C-terminal 6×His tagged protein (Cpe^Sc-6×His^) was also expressed in *S*. *clavuligerus* and *E*. *coli*. Engineered oligonucleotide primers were used to introduce a C-terminal 6×His tag during the amplification of *cpe*^*Sc*^ (S1 Table), which was cloned into pHM11a for expression in *S*. *clavuligerus*. For expressing Cpe^Sc-6×His^ in *E*. *coli*, the gene was PCR amplified using primers listed in S1 Table, was cloned into pET30b for expression at 15°C for 24 hours. Cpe^Sc-6×His^ protein was purified using Ni-NTA resin as per the manufacturer’s instructions (Qiagen, USA) and was stored in 20 mM Tris-HCl, 150 mM NaCl (pH 7.6) + 20% (v/v) glycerol.

For western analysis, 20–50 μg of cell-free extract or 0.5–1 μg of purified Cpe^Sc-6×His^ was subjected to standard 12% SDS-PAGE before being transferred to Immobilon-P PVDF membranes according to the manufacturer’s recommendations (Millipore, Canada). Membranes were washed with TBS-T buffer (50 mM Tris-HCl pH 7.6, 150 mM NaCl, and 0.5% v/v Tween-20) and were blocked overnight at 4°C in blocking buffer (TBS-T with 10% w/v non-fat milk). The membranes were probed using anti-FLAG or anti-6×His antibodies (Thermo Scientific Pierce, USA) at 1:500 final dilutions before being washed several times with TBS-T buffer. The secondary antibody (Thermo Scientific Pierce, USA) was added at 1:400 dilution in TBS-T buffer and the membranes were processed using the ECL Western Blot Substrate (Promega, USA) for imaging using a GE ImageQuant LAS 4000 Digital Imaging System (GE Healthcare, USA).

### RNA isolation and RT-PCR analysis

*S*. *clavuligerus* wt and *Δcpe-INF* strains were used to isolate RNA after 48 hours of growth in SA medium using the innuSPEED Bacteria/Fungi RNA Kit and a bead beater as per the manufacturer’s instructions (Analytik Jena, Germany). The cDNA was synthesized using 500 ng of DNaseI-treated RNA using random hexameric primers provided with the SuperScript II reverse transcriptase (RT) kit as per the manufacturer’s recommendations (Invitrogen, USA). PCR was performed using 2.5μl of the RT product from above in a final volume of 20μl using the GoTaq DNA Polymerase (Promega, Canada). Thirty cycle PCR was performed to detect *ceaS2*, *oat2*, *oppA1*, *claR*, *car*, *cyp*, *cpe (orf12)*, *orf13*, *orf14*, *oppA2*, *orf16*, *gcas*, *pbpA*, and *hrdB* cDNA using gene-specific primers (S2 Table). Control reactions contained DNaseI-treated RNA preparations without reverse transcription for each reaction.

### Site-directed mutagenesis of Cpe^Sc^

The *cpe*^*Sc*^ gene along with the *ermE*p* from pHM11a was isolated as a BglII/BamHI fragment and inserted into the BamHI site of pSET152 (45) to prepare a smaller expression plasmid (pSET:*cpe*^*Sc*^), which would be more amenable for site-directed mutagenesis (Table 2). The QuikChange II Site-Directed Mutagenesis Kit (Agilent Technologies, USA) along with mutagenic oligonucleotide primers (S3 Table) and pSET:*cpe*^*Sc*^ as template was to prepare selected single amino acid variants of Cpe^Sc^ according to the manufacturer’s instructions. All introduced mutations were verified by DNA sequencing, and plasmids expressing Cpe^Sc^ variants (Table 2) were introduced into the *S*. *clavuligerus Δcpe-INF* mutant for complementation studies.

### Metabolite detection and analysis

*S*. *clavuligerus* strains were grown for fermentation studies and culture supernatants were assessed for clavulanic acid production using bioassays as described previously [32]. High-performance liquid chromatography analysis of imidazole-derivatized culture supernatants was performed using a 1260 Infinity system (Agilent Technologies, USA) and a Bondclone C18 (100×8mm, 10μm, 148Å) column (Phenomenex, USA) [23]. Selected supernatants were also analyzed by liquid chromatography-mass spectrometry on an LC-MS-Trap system (1100 LC-MS Agilent Technologies, USA) as previously described [23, 38], with the exception that an Xterra (2.1×150 mm, 3.5μm, 125Å) column (Waters Scientific, USA) was used in the analysis.

## Results

### Preparation and complementation of the *S*. *clavuligerus Δcpe*::*apra* and *Δcpe-INF* deletion mutants

In *S*. *clavuligerus*, *cpe* (*orf12*) and *orf13* are transcribed together as a polycistronic mRNA and the stop codon of *cpe* also overlaps with the start codon of *orf13* [23]. In addition, there is a 48 bp overlap between the 3' ends of *orf13* and *orf14*, which are encoded on opposite DNA strands (Fig 2A). Therefore, there is potential for polar effects on the expression (transcription and/or translation) of *orf13* in a *cpe* gene mutant, depending on how it was prepared. To test this hypothesis, two different *cpe* mutants (Table 1) were prepared using the ReDirect two-step protocol [34]. In the first mutant, the apramycin (*apra*) cassette flanked by FLP recombinase target (FRT) sites from the plasmid pIJ773 was used to delete an internal region of *cpe* (39 bp from the 5' end to 39 bp from the 3' end), leading to the *S*. *clavuligerus Δcpe*::*apra* mutant (Fig 2B). The *apra* gene was inserted in the orientation opposite to *cpe* transcription to maximize the potential for polar effects on the expression of downstream genes. For preparing the second mutant, the *apra* cassette was excised from the *Δcpe*::*apra* mutant and replaced with an 81 bp scar sequence in the correct reading frame to give the *S*. *clavuligerus Δcpe-INF* (in frame deletion) mutant (Fig 2B), which has the least potential for producing polar effects on the expression of the downstream genes. The prepared mutants were verified by genomic DNA PCR and were complemented using the *cpe* gene from *S*. *clavuligerus* (*cpe*^*Sc*^) expressed under the control of the constitutive ermE* promoter (*ermE*p*) in the plasmid pHM11a (Table 2). Wild-type and *cpe* mutant strains of *S*. *clavuligerus* containing either pHM11a (control) or pHM:*cpe*^*Sc*^ were grown in SA medium for up to 120 hours to assess for clavulanic acid production. Bioassays and HPLC analysis of culture supernatants demonstrated that both the *S*. *clavuligerus Δcpe*::*apra* and *Δcpe-INF* mutants were completely blocked in clavulanic acid production when compared to the wt strain (Fig 2C). Introduction of pHM:*cpe*^*Sc*^ restored clavulanic acid production to 60%-70% of wt levels in both mutants (Fig 2D), suggesting that the *cpe* disruption(s) was not associated with any significant polarity. The marker-less *S*. *clavuligerus Δcpe-INF* mutant was chosen for further analysis in the current study.

### Cellular localization of Cpe^Sc^ and its influence on the expression of other genes from the clavulanic acid biosynthetic gene cluster of *S*. *clavuligerus*

**Fig 3:**
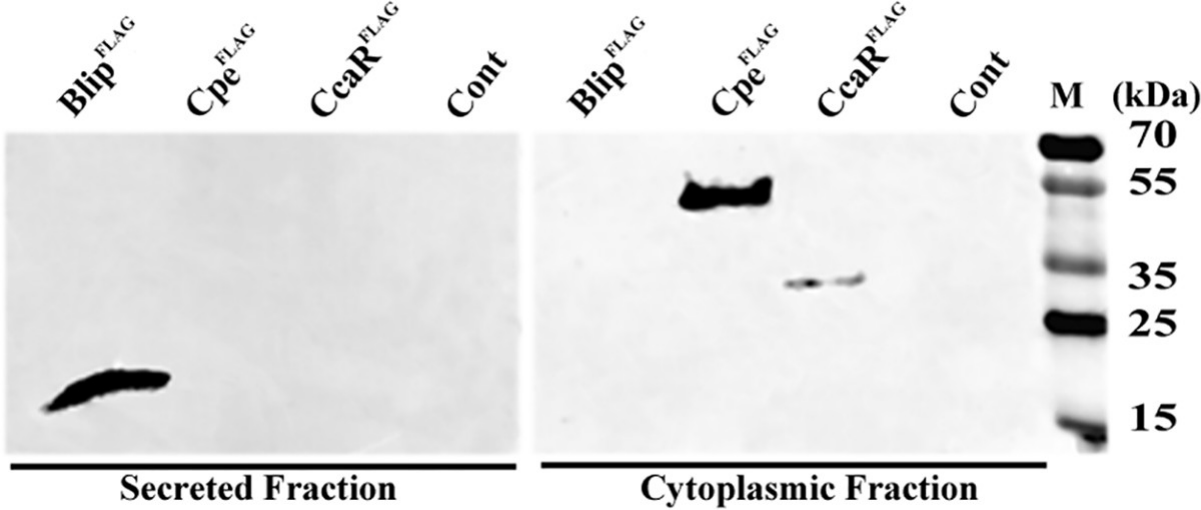
Cellular localization of Cpe in *S*. *clavuligerus*. C-terminal FLAG-tagged copies of Cpe, secreted Blip and cytoplasmic CcaR were expressed in wt *S*. *clavuligerus* separately for western blot analysis. Cultures were used for isolating secreted (left panel) and cell/cytoplasmic (right panel) fractions, which were probed using anti-FLAG antibodies. The analysis of protein fractions from *S*. *clavuligerus* strains containing plasmids pHM:*blip*^FLAG^ (expressing Blip^FLAG^), pHM:*cpe*^FLAG^ (expressing Cpe^FLAG^), pHM:*ccaR*^FLAG^ (expressing CcaR^FLAG^) or pHM11a (Cont, empty vector) is shown. Lane M contains the PageRuler Plus Prestained Protein Ladder, which functioned as the molecular weight marker for resolving protein samples during 12% SDS-PAGE.

The Cpe^Sc^ protein shares many sequence and structural similarities with class A β-lactamases, but it has been shown to lack any detectable β-lactamase activity under *in vitro* conditions [25]. Most bona fide β-lactamases are secreted proteins that inactivate β-lactam antibiotics in the periplasm, the site of peptidoglycan biosynthesis and crosslinking [39]. However, the predicted amino acid sequence of Cpe^Sc^ does not contain any detectable secretion signals, warranting further investigation into its exact cellular location in *S*. *clavuligerus*. C-terminal FLAG (Cpe^Sc-FLAG^) and 6×His (Cpe^Sc-6×His^) epitope-tagged copies of the protein were separately expressed in wt *S*. *clavuligerus* using the constitutive *ermE*p* from plasmid pHM11a (Table 2). As controls for protein localization studies, *S*. *clavuligerus* strains expressing C-terminal FLAG-tagged copies of known cytoplasmic (CcaR^FLAG^) [40] and secreted (Blip^FLAG^) [41] proteins were also prepared separately (Table 2). *S*. *clavuligerus* strains expressing FLAG-tagged copies of the respective proteins were grown in SA medium for 48 hours for isolating different cellular protein fractions. Mycelial pellets were used to obtain cytoplasmic and cell wall-associated fractions, whereas enriched secreted fractions were prepared by using salt to precipitate soluble proteins from culture supernatants. Western blot analysis of different cellular fractions using anti-FLAG polyclonal antibodies demonstrated that Cpe^Sc-FLAG^ was only detected in the cytoplasmic fraction (Fig 3). As expected, the CcarR^FLAG^ and Blip^FLAG^ controls were detected in cytoplasmic and secreted fractions, respectively (Fig 3). In addition, cultures of *S*. *clavuligerus* expressing Cpe^Sc-6×His^ were also used for isolating fractions for western analysis, which confirmed that Cpe^Sc^ is a cytoplasmic protein (S1 Fig). During the described western blot analysis, the size of epitope tagged Cpe^Sc^ was determined to be ~54 kDa based on the signal obtained using anti-FLAG and anti-6×His antibodies (S1 Fig). This corresponded to the size of 6×His-tagged Cpe^Sc^ heterologously expressed and purified from *E*. *coli*, which was used as a control (S1 Fig).

**Fig 4:**
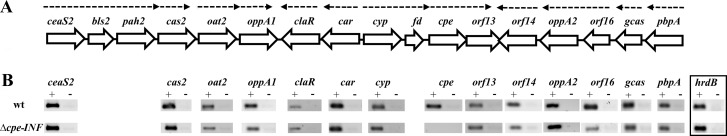
Transcriptional analysis of genes from the clavulanic acid biosynthetic gene cluster (BGC) in wt *S*. *clavuligerus* and the *Δcpe-INF* mutant. (A) The overall architecture of the BGC is shown with each hollow arrow representing a gene and the arrowhead its orientation. The known transcriptional units are also indicated, and the broken lines represent transcripts (B) The first gene from each transcriptional unit in (A) was selected for analysis to determine its comparative expression level in the two respective strains. RNA isolated from wt *S*. *clavuligerus* and the *Δcpe-INF* mutant after 48 hours of growth in SA medium was used for RT-PCR (+) analysis. As controls, treated RNA samples were used directly in PCR without RT or cDNA synthesis (-). The expression of the constitutively expressed *hrdB* gene (extreme right boxed panel) was used as internal control to normalize expression levels between different samples/strains.

Clavulanic acid has been shown to bind non-covalently with Cpe^Sc^ under *in vitro* conditions [25], but the relevance of this interaction is still not clear as the protein did not catalyze any associated reaction. In addition, Cpe^Sc^ is located in the cytoplasm of *S*. *clavuligerus* (Fig 3), and *cpe* mutants are completely blocked in clavulanic acid production (Fig 2C). This raised the possibility that the protein could have a role in functioning as a cytoplasmic sensor/receptor for clavulanic acid or related metabolites to indirectly regulate production under *in vivo* conditions. To test this hypothesis, we analyzed the expression level of the first gene from each transcriptional unit (Fig 4A) from the clavulanic acid gene cluster of *S*. *clavuligerus* in the *Δcpe-INF* mutant and compared it with that from the wt strain (Fig 4B). RT-PCR analysis showed that only expression of the *cpe* gene was altered in the comparison, which was expected (Fig 4B). The analysis also demonstrated that the *Δcpe-INF* mutation is not associated with any transcriptional polarity as the expression of *orf13* was unaffected in the strain. Therefore, it is clear that the deletion of *cpe* does not in any way influence the expression of other genes from the clavulanic acid gene cluster in *S*. *clavuligerus*.

### Assessing the requirement of the N- and C-terminal domains of Cpe^Sc^ for clavulanic acid production in *S*. *clavuligerus*

**Fig 5:**
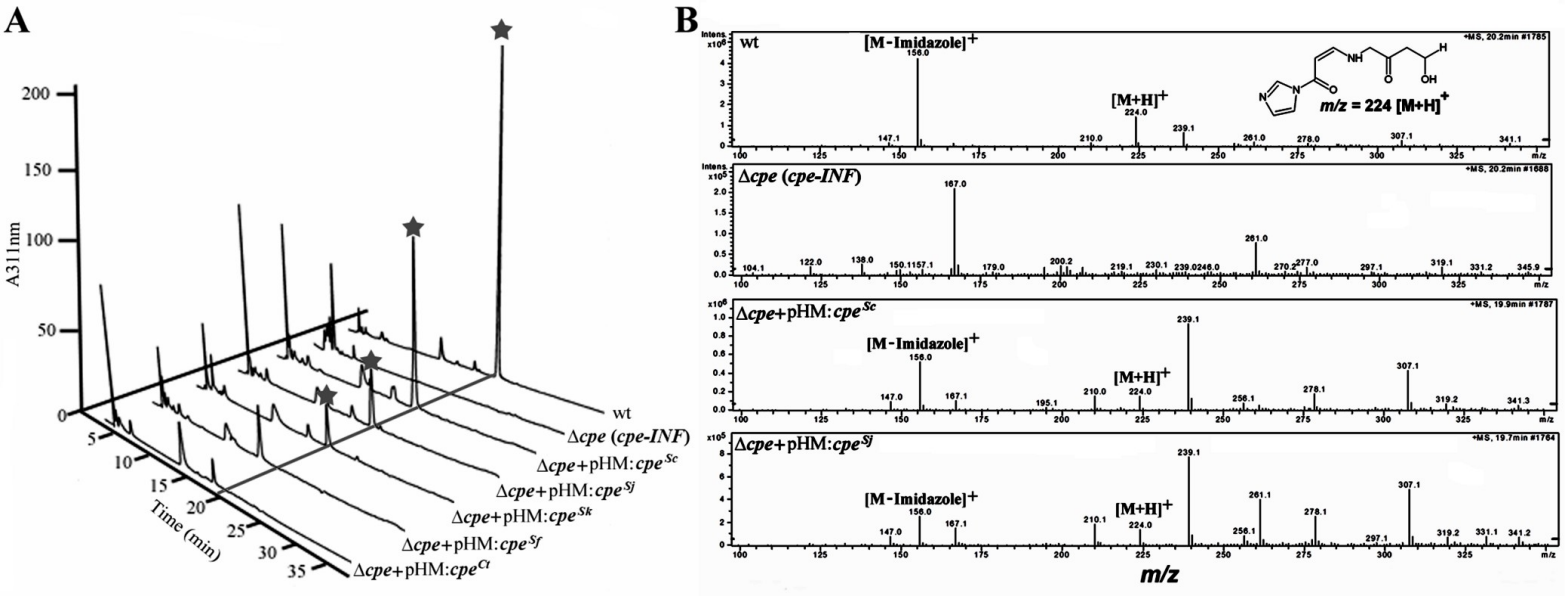
Functional analysis of different domains of Cpe^Sc^ and its homologues during clavulanic acid production in *S*. *clavuligerus*. (A and B) LC-MS analysis of 96 hour SA culture supernatants after imidazole derivatization using the ammonium bicarbonate buffer system [38]. Cultures of wt *S*. *clavuligerus* or the *Δcpe*-*INF* mutant expressing Cpe from *S*. *clavuligerus* (pHM:*cpe*^*Sc*^), *S*. *flavogriseus* (pHM:*cpe*^*Sf*^), *S*. *jumonjinensis*/*S*. *katsurahamanus* (pHM:*cpe*^*Sj/Sk*^) or the C-terminal domain of Cpe^Sc^ (pHM:*cpe*^*Ct*^) were used in the analysis. (A) Liquid chromatography profiles showing the elution of the peaks corresponding to imidazole-derivatized clavulanic acid (indicated by the star symbol). (B) Mass spectra of the major peaks corresponding imidazole-derivatized clavulanic acid [M+H]^+^ (m/z = 224) and the fragmented product [M-imidazole]^+^ (m/z = 156), which were only detected in supernatants from clavulanic acid producing strains shown in (A).

The crystal structure of heterologously expressed Cpe^Sc^ from *E*. *coli* demonstrated that it contains distinct N-terminal (residues 1–127) and C-terminal (residues 128–458) domains resembling ketosteroid isomerases/polyketide cyclases and β-lactamases, respectively [25]. It was also shown that the C-terminal domain was responsible for the observed *in vitro* cephalosporin esterase activity of Cpe^Sc^, but a function or phenotype could not be assigned at the time for the N-terminal domain based on the assays used [25]. Results from the western blot analysis described above indicate that Cpe^Sc^ does not undergo posttranslational processed in *S*. *clavuligerus*, but it is not known if the N-terminal domain is required for the activity of the protein in the native host. To investigate the *in vivo* roles of the N- and C-terminal domains during clavulanic acid biosynthesis, three additional Cpe^Sc^ expression constructs were prepared for analysis. The N- and C-terminal domains were expressed separately or together (as separate polypeptides, Table 2) in complementation studies using the *S*. *clavuligerus Δcpe-INF* mutant. Analysis of SA and soy culture supernatants showed that except for full-length Cpe^Sc^, none of the other expression plasmids restored clavulanic acid production in the *Δcpe-INF* mutant, suggesting that both domains need to be part of a single polypeptide for biosynthesis to occur (Fig 5A and S2 Fig). Since the 5*S* clavams are not produced by wt *S*. *clavuligerus* when grown in SA medium [42], soy cultures were included in the analysis. Results showed that none of the strains accumulated any of the known intermediates from the clavulanic acid arm of the pathway (Fig 1 and S4 Table), and production of the 5*S* clavams was also unaffected in all of them when cultured in soy medium (S5 Table).

### Examination of the ability of other *cpe* homologues to support clavulanic acid production in the *S*. *clavuligerus Δcpe-INF* mutant

The *S*. *flavogriseus* and *S*. *viridis* genome sequences revealed that they encode clavulanic-like BGCs [13], which are thought to be “silent or cryptic” as the two organisms are not known to produce any clavam metabolites [15]. Whereas other studies have shown that *S*. *jumonjinensis* and *S*. *katsurahamanus* can also produce clavulanic acid [12], but details regarding the sequences of their respective gene clusters are unavailable [14]. Therefore, we amplified the *cpe* homologues from the four organisms using genomic DNA as a template for complementation studies, and we also determined the complete sequence of the genes from *S*. *jumonjinensis* and *S*. *katsurahamanus* (S3 Fig). The predicted amino acid sequences of the Cpe proteins from *S*. *jumonjinensis* (Cpe^Sj^) and *S*. *katsurahamanus* (Cpe^Sk^) share ~68% identity with Cpe from *S*. *clavuligerus* (Cpe^Sc^) (S4 Fig). In comparison, the predicted sequences of the proteins from *S*. *flavogriseus* (Cpe^Sf^) and *S*. *viridis* (Cpe^Sv^) showed 58.8% and 48.8% identity to Cpe^Sc^, respectively. The predicted C-terminal domains of all four proteins contain the characteristic class A β-lactamase SXXK and SDN catalytic motifs, whereas the KTG motif was replaced by KGG in the non-producers (*S*. *flavogriseus* and *S*. *viridis*) and KAG in the producers (*S*. *clavuligerus*, *S*. *jumonjinensis* and *S*. *katsurahamanus*), respectively (S4 Fig). In addition, all proteins also contained an extra N-terminal domain resembling that of Cpe^Sc^ to different extents.

Sequence analysis showed that the Cpe proteins from clavulanic acid producers are more closely related to each other as compared to those from the non-producers (S4 and S5 Figs). To determine the significance of this finding, the respective *cpe* genes from different sources were expressed under the control of *ermE*p* in the *S*. *clavuligerus Δcpe-INF* mutant for complementation studies (Table 2). It was found that Cpe from *S*. *jumonjinensis* and *S*. *katsurahamanus* restored clavulanic acid production in the *S*. *clavuligerus Δcpe-INF* mutant to varying degrees (Fig 5A and 5B), whereas no complementation was observed in the case of Cpe^Sv^ and Cpe^Sf^. Therefore, only the *cpe* genes from clavulanic acid producers (Cpe^Sj^ and Cpe^Sk^) seem to be functionally equivalent to the known homologue from *S*. *clavuligerus* during clavulanic acid biosynthesis.

### Identification of amino acid residues from Cpe^Sc^ required for clavulanic acid production in *S*. *clavuligerus*

The crystal structure of Cpe^Sc^ revealed that two molecules of clavulanic acid (CA-1 and CA-2, respectively) bind to the monomeric protein [25]. CA-1 binds to an active site pocket to form hydrogen bonds with Lys_89_, Tyr_359_ and Arg_418_ and its C2 side chain carboxylate is positioned deep in the active site of Cpe^Sc^, where it interacts with Lys_375_ [25]. Lys_375_ is part of the Cpe^Sc^ KTG motif, where the equivalent catalytic residues in PBPs/β-lactamases also interact with the analogous carboxylates from penicillin and cephalosporin substrates, respectively [43]. In comparison, binding of CA-2 occurs in a mostly hydrophobic cleft comprised of Trp_91_, Leu_362_, Leu_415_, Arg_418_ and Ala_422_ at the interface of the N- and C-terminal domains [25]. The Trp_91_ and Arg_418_ residues are also highly conserved in the other predicted Cpe proteins (Cpe^Sj^, Cpe^Sk^, Cpe^Sf^, Cpe^Sv^), where Arg_418_ from Cpe^Sc^ is also involved in binding to CA-1 (S4 Fig). Therefore, Lys_89_, Trp_91_ Tyr_359_, Lys_375_ and Arg_418_ from Cpe^Sc^ were selected for mutagenesis studies to examine their *in vivo* contributions during the clavulanic acid production in *S*. *clavuligerus*. The *cpe*^*Sc*^ gene along with the *ermE*p* was transferred from pHM:*cpe*^*Sc*^ to pSET152 and subjected to site-directed mutagenesis, and the prepared *cpe*^*Sc*^ variants were assessed for their ability to complement the *S*. *clavuligerus Δcpe-INF* mutant. Replacement of Lys_89_, Tyr_359_, Lys_375_ or Arg_418_ with Ala individually in Cpe^Sc^ led to a complete loss in clavulanic acid production (Table 3 and S6 Fig). However, when Lys_375_ was replaced with arginine (both being basic amino acids), clavulanic acid production was restored to 40% production levels in the *Δcpe-INF* mutant as compared to the wt strain (Table 3 and S6 Fig). As well, partial complementation was also observed in the case of the Cpe^Sc^ Trp_91_Ala variant (Table 3).

**Table 3:** Clavulanic acid production in wild type (wt) *S*. *clavuligerus* and the *Δcpe*-*INF* mutant expressing different variants of Cpe^Sc^.

*S*. *clavuligerus* strain^a^	Cpe^Sc^ protein variant^b^ (*cpe*^*Sc*^ codon substitution)	Bioactivity^c^	
		SA	soy
wt	NA	**++++**	**+++++**
*Δcpe-INF*	NA	-	-
*Δcpe-INF* (pSET-152)	NA	**-**	**-**
*Δcpe-INF* (pSET:*cpe*^*Sc*^)	wt (none)	**+++**	**++++**
*Δcpe-INF* (pSET:*cpe*^*Sc-* Ser27Ala^)	Ser_27_Ala (TCC→GCC)	**+++**	**++++**
*Δcpe-INF* (pSET:*cpe*^*Sc****-***Lys89Ala^)	Lys_89_Ala (AAG→GCG)	**-**	**-**
*Δcpe-INF* (pSET:*cpe*^*Sc*-Trp91Ala^)	Trp_91_Ala (TGG→GCG)	**++**	**+++**
*Δcpe-INF* (pSET:*cpe*^*Sc-*Arg115Ala^)	Arg_115_Ala (CGC→GCC)	**+++**	**++++**
*Δcpe-INF* (pSET:*cpe*^*Sc-*Ser173Ala^)	Ser_173_Ala (TCG→GCG)	**-**	**-**
*Δcpe-INF* (pSET:*cpe*^*Sc-*Lys176Ala^)	Lys_176_Ala (AAG→GCG)	**-**	**-**
*Δcpe-INF* (pSET:*cpe*^*Sc*-Ser206Ala^)	Ser_206_Ala (AGC→GCC)	**+++**	**++++**
*Δcpe-INF* (pSET:*cpe*^*Sc*-Ser234Ala^)	Ser_234_Ala (AGC→GCC)	**-**	**-**
*Δcpe*-INF (pSET:*cpe*^*Sc*-Arg311Ala^)	Arg_311_Ala (CGC→GCC)	**+++**	**++++**
*Δcpe-INF* (pSET:*cpe*^*Sc*-Gln321Ala^)	Gln_321_Ala (CAG→GCG)	**+++**	**++++**
*Δcpe-INF* (pSET:*cpe*^*Sc*-Trp326Ala^)	Trp_326_Ala (TGG→GCG)	**+++**	**++++**
*Δcpe-INF* (pSET:*cpe*^*Sc*-Arg346Ala^)	Arg_346_Ala (CGG→GCG)	**+++**	**++++**
*Δcpe-INF* (pSET:*cpe*^*Sc*-Tyr359Ala^)	Tyr_359_Ala (TAC→GCC)	**-**	**-**
*Δcpe-INF* (pSET:*cpe*^*Sc*-Lys375Ala^)	Lys_375_Ala (AAG→GCG)	**-**	**-**
*Δcpe-INF* (pSET:*cpe*^*Sc*-Lys375Arg^)	Lys_375_Arg (AAG→AGG)	**++**	**+++**
*Δcpe-INF* (pSET:*cpe*^*Sc*-Ser378Ala^)	Ser_378_Ala (TCC→GCC)	**-**	**-**
*Δcpe-INF* (pSET:*cpe*^*Sc-*Arg418Ala^)	Arg_418_Ala (CGC→GCC)	**-**	**-**

^a^ Strains of *S*. *clavuligerus* were fermented in either SA or soy media for 96 hours and culture supernatants were used in bioassays for detecting clavulanic acid production.

^b^ Single amino acid variants of Cpe^Sc^ used in the analysis are shown and the corresponding codon changes in *cpe*^*Sc*^ leading to the respective substitutions are indicated in parenthesis; NA, Not applicable.

^c^ Zones of inhibition relative the wt strain grown in each media are indicated, where (+) indicates clavulanic acid production and (-) indicates the lack of production, respectively.

Other amino acids from Cpe^Sc^ have also been shown to interact with clavulanic acid, some of which contributed to its *in vitro* cephalosporin esterase activity [25]. These include residues from the SXXK (Ser_173_) and SDN (Ser_234_) motifs comprising the catalytic tetrad (Ser_173_/Lys_176_/Ser_234_/Lys_375_), which is conserved in all four Cpe protein sequences described above (S4 Fig). Valegård, et al. reported that the Cpe^Sc^ Ser_173_Ala mutant showed a 100-fold reduction in esterase activity, whereas the Ser_234_Ala and Ser_378_A mutants were not affected to the same extent [25]. Since the roles of the respective amino acids during clavulanic acid are not known, Ser_173_, Lys_176_, Ser_234_, Ser_378_ were also individually substituted with Ala in Cpe^Sc^ for *in vivo* analysis. All four variants were unable to complement the *Δcpe-INF* mutant, demonstrating that they essential for clavulanic acid production in *S*. *clavuligerus* (Table 3 and S6 Fig).

In the current analysis, amino acids were also identified that are either highly and/or partially conserved in all five Cpe proteins (S4 Fig). These include the Ser_27_, Arg_115_, Ser_206_, Arg_311_, Gln_321_, Trp_326_ and Arg_346_ from Cpe^Sc^, which are not part of any conserved motif and do not interact with clavulanic acid directly based on the reported crystal structure of the protein (29). When each of these residues was replaced with Ala, the respective Cpe^Sc^ variants restored clavulanic acid production in the *S*. *clavuligerus Δcpe-INF* mutant to varying degrees (Table 3), demonstrating that they are not essential for production. Overall, a detailed set of residues were identified in Cpe^Sc^, some of which contribute to both the *in vitro* and *in vivo* activities of the protein, whereas others are only relevant during the latter process. These are important findings as they allude to the actual biochemical role/function of the protein, which occurs during *in vivo* clavulanic acid production in *S*. *clavuligerus*

## Discussion

In the current study, we examined the function of *cpe* from the CA-BGC of *S*. *clavuligerus*, starting with the significance of the relative arrangement of neighboring genes located in its immediate vicinity. Polycistronic mRNAs often allow for the concerted expression of gene products involved in related biosynthetic pathways [44, 45], and gene knockout studies have implicated *cpe* as being essential for clavulanic acid production in *S*. *clavuligerus* [21, 23]. *cpe* is transcribed as part of a bicistronic operon along with *orf13*, the start codon of which also overlaps with the stop codon of *cpe* (Fig 2A), suggesting potential co-translation [46–48]. In addition, the 3′ ends of *orf13* and *orf14* overlap (Fig 2A), which is unusual in bacteria [49]. It can be challenging to decipher the precise roles of genes located within operons, particularly in cases where co-translation is involved [50–53]. The disruption of genes located in the 5′ regions of operons can influence the expression of downstream genes and also impact the relative stoichiometry of encoded gene products, thereby leading to polar effects [47, 51, 54]. Therefore, we prepared an in-frame *S*. *clavuligerus cpe* deletion mutant for use in the current study, while maintaining its stop codon and context with *orf13* to minimize the potential for polar effects. During the process, we also prepared the *S*. *clavuligerus Δcpe*::*apra* deletion mutant in which a disruption cassette was inserted in the opposite orientation to *cpe* transcription. It was noted that both the in-frame and the insertional mutant could be successfully complemented to restore clavulanic acid production using a plasmid-borne copy of *cpe*, demonstrating that polar effects were not associated with either of them. Therefore, it seems that despite their relative organization, alternate mechanisms exist to facilitate the translation of Orf13 in the *cpe* mutants, enabling us to use the in-frame mutant for more detailed *in vivo* studies.

The Cpe protein resembles class A β-lactamases [25], proteins which are secreted to inactivate β-lactam antibiotics before they can inhibit peptidoglycan crosslinking on the outer surface of the cytoplasmic membrane. In addition, when *cpe* was first sequenced it was reported to share some similarity with the LpqF lipoprotein from *Mycobacterium tuberculosis*, [25], the function of which is still unknown [55]. Most β-lactamases are secreted using the Sec pathway [39], however, some are translocated by the Tat system in mycobacteria [56]. In addition, certain proteins unrelated to β-lactamases have been reported to be secreted in the absence of any recognizable N-terminal signal sequences [57]. To narrow down its biological function, we investigated the cellular location of Cpe^Sc^ in its native host and showed that it is a cytoplasmic protein, with no evidence of any association with the cell wall or secreted fractions. Therefore, it can be inferred that unlike β-lactamases, the *in vivo* role of Cpe^Sc^ lies in the cytoplasm, which is also the site of clavulanic acid biosynthesis in *S*. *clavuligerus*.

During the biosynthesis of certain bioactive natural products, mechanisms exist to coordinate different stages involved in the production of the terminal metabolite [58]. The strategy is used to regulate the expression of specific genes, including those involved in export or self-resistance, when threshold concentrations of a specific intermediate(s) from the pathway is achieved in the cell [59, 60]. As well, feedback inhibition during natural product biosynthesis by end products is also well documented [61]. The cytoplasmic location of Cpe^Sc^ and its ability to bind to certain cephalosporins and clavulanic acid raises the possibility that it could function as an intracellular receptor for sensing such metabolites to elicit an associated response directly or indirectly. For example, the membrane-associated sensor kinase BlaR from *Staphylococcus aureus* also contains a PBP-like domain (related to β-lactamases) that binds to β-lactams and triggers the proteolysis of the cytoplasmic BlaI repressor to activate the expression of the BlaZ β-lactamase [62]. To investigate this hypothesis, the expression of key transcriptional units (comprising *ceaS2*, *oat2*, *oppA1*, *claR*, *car*, *cyp*, *cpe*, *orf13*, *orf14*, *orf16*, *gcas* and *pbpA*) from the CA-BGC was analyzed in the *S*. *clavuligerus Δcpe-INF* mutant for comparison with the wt strain. Except for the expression of *cpe* itself, the transcription of all other analyzed genes was unaffected in the mutant (Fig 4B). Results also clearly demonstrated that the transcription of *orf13* (and *orf14*) was not affected in the *Δcpe-INF* mutant, despite their complex transcriptional/transnational arrangement (Fig 2A). Therefore, based on the results, we can rule out any apparent sensory or regulatory role for Cpe^Sc^ during clavulanic acid biosynthesis in *S*. *clavuligerus*.

Homologues of *cpe*^*Sc*^ are also present in related BGCs from other clavulanic acid producing (*S*. *jumonjinensis* and *S*. *katsurahamanus*) and non-producing (*S*. *flavogriseus* and *S*. *viridis*) organisms (S4 Fig). It was found that only expression of Cpe from producer species (Cpe^Sj^/Cpe^Sk^) could complement the *S*. *clavuligerus Δcpe-INF* mutant. The probability of two proteins having a similar biological function increases proportionally with the relatedness of their respective amino acid sequences [63]. This might explain the complementation phenotypes as Cpe^Sj^/Cpe^Sk^ are more closely related to Cpe^Sc^ than Cpe^Sf^/Cpe^Sv^ (S4 and S5 Figs). The inability of the corresponding *S*. *flavogriseus* and *S*. *viridis cpe* homologues to complement the *S*. *clavuligerus* mutant suggests that portions of the respective clavulanic acid-like BGCs from the non-producers (including *cpe*) might also be defective in addition to being “silent” [15], a hypothesis that is currently being explored. Since the same plasmid(s) and constitutive promoter (*ermE*p*) was used to drive the expression of all *cpe* genes independently in the current study, the lack of observed complementation in some cases is unlikely due to differences in expression levels.

A previous study showed that the C-terminal domain of recombinant Cpe^Sc^ displays *in vitro* O-acetyl cephalosporin esterase activity, but a function could not be assigned for its N-terminal domain [25]. It was also suggested that Cpe^Sc^ might undergo *in vivo* post-translational processing in *S*. *clavuligerus* to separate the two domains, which could not be addressed at the time since the protein was heterologous expressed and purified from *E*. *coli* [25]. Therefore, we examined the requirement of the two Cpe^Sc^ domains during *in vivo* clavulanic acid production in *S*. *clavuligerus* by using them to complement the *Δcpe-INF* mutant. Results suggest that both domains are required, and that they should be present to on a single peptide for production to take place (S2 Fig). It is also possible that the inclusion of the two domains on separate plasmid constructs could lead to reduced expression of the respective peptides or they could become unstable/misfolded, which might explain the lack of complementation. This seems unlikely, as the C-terminal domain of Cpe^Sc^ (completely lacking the N-terminus) was previously expressed and purified from *E*. *coli* for biochemical and structural studies [25]. Therefore the inability of the Cpe^Sc^ C-terminal domain to complement the *S*. *clavuligerus Δcpe-INF* mutant is most likely due to the missing region of the protein, which is the N-terminus isomerase like domain. We also show that Cpe^Sc^ with specific amino acid substitutions (but not all) in either its N- or C-terminal domain is unable to complement the *Δcpe-INF* mutant and that only a single band corresponding to intact Cpe^Sc^ was observed in protein fractions from *S*. *clavuligerus* during western analysis (Fig 3 and S1 Fig). Therefore, results clearly demonstrate that Cpe^Sc^ does not undergo of *in vivo* proteolytic processing in *S*. *clavuligerus* and that its N-terminal isomerase-like domain is required for clavulanic acid production, which is the first direct evidence for its involvement in the process.

The crystal structure of Cpe^Sc^ showed that the protein binds to two molecules of clavulanic acid [25]. The first molecule (CA-1) forms hydrogen bonds with Tyr_359_, Arg_418_ and Lys_89_ from the active site, which also contains Ser_173_ and Ser_234_ from the S_173_XXK_176_ and S_234_DN motifs, respectively. In addition, these 5 amino acids are conserved across all five Cpe homologues included in the current study (S4 Fig). Ser_173_, Ser_234_ and Ser_378_ from Cpe^Sc^ are also important for the *in vitro* cephalosporin esterase activity of the protein [25], whereas in class A β-lactamases the equivalent residues (including Lys_176_) are required for the binding and acylation of β-lactam substrates during catalysis [64–66]. It has been reported that certain esterases also exhibit β-lactamase activity and that some PBPs can conversely function as esterases [67, 68], which is not a true representation of their actual physiological function(s). This could also be the case for Cpe^Sc^, where the protein can function as an esterase if given permissive substrates (25). In the current study, we demonstrated that Ser_173_, Lys_176_, Ser_234_ and Ser_378_ from Cpe^Sc^ are essential for *in vivo* clavulanic acid production in *S*. *clavuligerus*, reminiscent of their catalytic roles in class A β-lactamases. The K_234_ (T/S)_235_G catalytic motifs of serine β-lactamases also contain a conserved Ser/Thr residue, which interacts with the carboxylates of corresponding β-lactam substrates [69, 70]. Substitution of this Ser/Thr by amino acids with non-hydroxylated side chains (such as Ala) significantly reduces β-lactamase activity, especially against cephalosporin substrates [70, 71]. It is interesting to note that this conserved Ser/Thr residue is replaced by Gly (KGG) or Ala (KAG) in Cpe from clavulanic acid non-producers and producers, respectively (S4 Fig), which might explain why Cpe^Sc^ lacks any detectable *in vitro* β-lactamase activity. As well, substitution of Lys_234_ by Thr (but not Arg) in class A β-lactamases substantially reduces their catalytic activities and ability to bind to clavulanic acid for inhibition [72, 73]. We also show that the positively charged electrostatic feature at position 375 is essential for the *in vivo* functional activity of Cpe^Sc^_,_ as replacement of Lys (K_375_TG) with Ala but not Arg in the protein completely abolished clavulanic acid production in *S*. *clavuligerus* (Table 3 and S6 Fig).

The *in vivo* role of amino acids from Cpe^Sc^ that interact with the second molecule of clavulanic acid (CA-2) *via* weak electrostatic interactions was also examined. These include the Trp_91_ or Arg_418_ residues [25], the substitution of which with Ala either reduced or abolish clavulanic acid production (Table 3). The blocked phenotype of the Arg_418_ mutant is consistent with the role of Arg_418_ as part of the CA-1 binding active site described above. In contrast, substitution of all other residues from the CA-2 binding cleft in Cpe^Sc^ did not significantly impact clavulanic acid production in *S*. *clavuligerus* (Table 3), suggesting that they do not contribute towards catalysis. Therefore, the role of the second clavulanic acid binding site in Cpe^Sc^ remains unclear. It is possible that the site occupied by CA-2 binds some other ligand and/or is an artifact of co-crystallizing purified Cpe^Sc^ with clavulanic acid during structural studies (86, 87), possibilities that warrant further examination. In addition, the substitution of other residues in Cpe^Sc^ that are conserved across all five homologues (S4 Fig), but which do not interact with clavulanic acid and/or are not part of any recognizable motif, did not affect the *in vivo* activity of the protein in *S*. *clavuligerus*.

To summarize, in the current study specific residues from Cpe^Sc^ and its two domains were shown to be essential for *in vivo* clavulanic acid production in *S*. *clavuligerus* (Table 3). These are novel finding and allude towards a biosynthetic role for the protein during production. The described Cpe proteins share many similarities with class A serine β-lactamases, but some crucial differences are also apparent. For example, class A β-lactamases possess the characteristic Ω loop containing residue(s) involved in deacylation and subsequent release of hydrolyzed substrates [74], which are missing in Cpe^Sc^. In comparison, class C and D serine β-lactamases also lack the Ω loop and are believed to use alternate mechanisms involving the SXXK and SDN motifs for deacylation instead [75]. The corresponding residues from Cpe^Sc^ (S_173_XXK and S_234_DN) bind to CA-1 in the crystal structure of the protein [25], but it is also possible that they might interact with some other intermediate(s) from the clavulanic acid biosynthetic pathway. Such precursors would not be detected in co-crystals reconstituted using heterologously expressed Cpe^Sc^ and purified clavulanic acid, as all other components of the biosynthetic pathway would be missing. Therefore, Ser_173_ and Ser_234_ could promote a nucleophilic attack on a still unknown substrate to form the primary Cpe-substrate complex, while other essential residues including some form the N-terminal domain (Lys_89_, Lys_375_ and Arg_418_) might be involved in stabilizing the intermediate followed by isomerization and product formation. The N-terminal domain of Cpe^Sc^ (residues 1–127) is structurally similar to a putative ketosteroid isomerase from *Shewanella frigidimarina*, which also contains the equivalent Lys_89_ from Cpe^Sc^ shown to interact with CA-1 [25]. Lys_89_ and some of its neighboring residues are conserved in all five Cpe homologues included in the current study (S4 Fig). For the first time, we show that the N-terminal domain of Cpe^Sc^ and specifically the Lys_89_ residue from it plays an essential role during clavulanic acid production in *S*. *clavuligerus* (S2 Fig and Table 3). We did not detect precursors or shunt products from the clavulanic acid pathway in culture supernatants from different *S*. *clavuligerus* mutants (S1 Table), suggesting that reaction intermediates remain tightly/covalently associated with Cpe^Sc^ during catalysis or are perhaps unstable [25]. Therefore, it is possible that Cpe^Sc^ is involved in an “altered/modified” β-lactamase-derived reaction required for clavulanic acid production by itself or in combination with another proteins(s), a hypothesis that is currently under investigation. It has been previously suggested that Cpe could be involved in the epimerization of 5*S* precursors to the 5*R* configuration during clavulanic acid biosynthesis [25], but this has not been demosntrated. Such a role for Cpe^Sc^ is conceivable based on the stereospecificity and reversible nature of enzyme-catalyzed reactions, but is not trivial to examine as the natural substrate(s) of Cpe are unknown [25]. It is intriguing that the biosynthetic pathway for a β-lactamase inhibitor (clavulanic acid) has recruited an enzyme for its production that is evolutionarily related to the very proteins that it inhibits. In the long term, deciphering the roles of different residues from Cpe^Sc^ involved in catalysis can enable us to engineer protein variants with the ability to accept altered substrates. Such a strategy would allow for the production of clavulanic acid analogues for future studies and possible applications.

## Supporting information

S1 Fig Western blot analysis of C-terminal 6×His tagged Cpe (Cpe^Sc-6×His^) expressed and purified from *E. coli* (using pET 30b-*cpe^Sc^*) or expressed in *Streptomyces clavuligerus* (using pHM:*cpe^Sc-6×his^*). Purified protein (*E*. *coli*) or cell free lysates (*S*. *clavuligerus*) were used in the analysis along with anti-6×His antibodies for detecting epitope-tagged Cpe. The lane labeled as “Mock prep” contains *S*. *clavuligerus* pHM11a empty vector lysate as control to account for any non-specific antibody binding. The size of the band corresponding to Cpe^Sc-6×His^ in all lanes was approximately 50–55 kDa, and the prestained protein ladder (Marker) was used as a reference for estimating molecular weights during 12% SDS-PAGE.
(PDF)

S2 Fig HPLC analysis of 96 hour SA culture supernatants from the *S. clavuligerus Δcpe-INF* strain expressing full-length Cpe^Sc^ (pHM:*cpe^Sc^*), its N-terminus (pHM:*cpe^Nt^*), C-terminus (pHM:*cpe^Ct^*) or both the N- and C-terminal domains at the same time as separate peptides (pHM:*cpe^Ct+Nt^*). The peak corresponding to imidazole-derivatized clavulanic acid (CA) is indicated and was only observed when the full-length protein was used in the analysis.
(PDF)

S3 Fig DNA sequences of *cpe* homologues from *S. jumonjinensis and S. katsurahamanus*. The complete gene sequences starting from initiation (ATG) to the stop (TGA) codon for each gene were determined as part of the current study and are reported.
(PDF)

S4 Fig Multiple sequence alignment of different Cpe proteins described in the current study. Analysis was performed with Clustal Omega (ver 1.2.1) using translated Cpe amino acid sequences from *S*. *clavuligerus* (WP_003952519.1), *S*. *flavogrisius* (WP_014152684.1), *S*. *viridis* (WP_015787620), *S*. *jumonjinensis* and *S*. *katsurahamanus*. The DNA sequences of *cpe* from the latter two producers (*S*. *jumonjinensis* and *S*. *katsurahamanus*) were determined as part of the current study and are reported in S3 Fig. The boxes in black highlight the conserved SXXK, SDN and KTG/KAG motifs present in class A β-lactamses and the respective Cpe proteins, whereas the box in blue represents the N-terminus domain indentifed in Cpe^Sc^. The arrows indicate amino acids from Cpe^Sc^ that were selected for mutagenesis and the ones highlighted in red were shown to be essential for *in vivo* clavulanic acid production in *S*. *clavuligerus*.
(PDF)

S5 Fig Phylogenetic relationship between select class A β-lactamases and Cpe proteins described in the current study. Multiple sequence alignments using the predicted amino acid sequences of Cpe proteins listed in S4 Fig. and class A β-lactamases including Bla (from the *S*. *clavuligerus* cephamycin C biosynthetic gene cluster, CAA90895.1) and TEM-1 (from *E*.*coli*, AMM70781.1) were used to prepare the tree, and bootstrap analyses were performed using 100 replicates. All positions containing gaps were eliminated during the analysis and the number next to each node represents the percentage of trees in which the respective topologies were observed.
(PDF)

S6 Fig HPLC analysis of 96 hour *S. clavuligerus* SA culture supernatants from the wt strain for comparison with the *Δcpe-INF* mutant expressing CpeSc or select single amino acid variants (Ser173Ala, Lys176Ala, Lys375Ala, Lys375Arg Ser234Ala) of the protein in trans using plasmid pHM11a. Clavulanic acid (CA) production was monitored at 311nm following imidazole derivatization.
(PDF)

S1 Table Oligonucleotide primers used for cloning and sequencing in the current study. (PDF)

S2 Table Gene specific primer pairs used for RT-PCR analysis in the current study. (PDF)

S3 Table Primer pairs used for site directed mutagenesis of *cpe*^Sc^. (PDF)

S4 Table LC-MS analysis of SA culture supernatants from wt *S. clavuligerus* and *Δcpe-INF* mutant strains for detecting clavulanic acid and pathway intermediates. (PDF)

S5 Table LC-MS analysis of soy medium culture supernatants from wt *S. clavuligerus* and *Δcpe-INF* mutant strains for detecting 5*S* clavam production. (PDF)
