# Supplementary material for: In vivo functional analysis of a class A β-lactamase-related protein essential for clavulanic acid biosynthesis in Streptomyces clavuligerus
Source: PLoS One. 2019 Apr 23;14(4):e0215960. doi: 10.1371/journal.pone.0215960 (PMC6478378; doi:10.1371/journal.pone.0215960)
Supplement: S1 Fig — Purified protein (E. coli) or cell free lysates (S. clavuligerus) were used in the analysis along with anti-6×His antibodies for detecting epitope-tagged Cpe. The lane labeled as “Mock prep” contains S. clavuligerus pHM11a empty vector lysate as control to account for any non-specific antibody binding. The size of the band corresponding to CpeSc-6×His in all lanes was approximately 50–55 kDa, and the prestained protein ladder (Marker) was used as a reference for estimating molecular weights during 12% SDS-PAGE. (PDF) [file pone.0215960.s001.pdf]

S1 Fig.

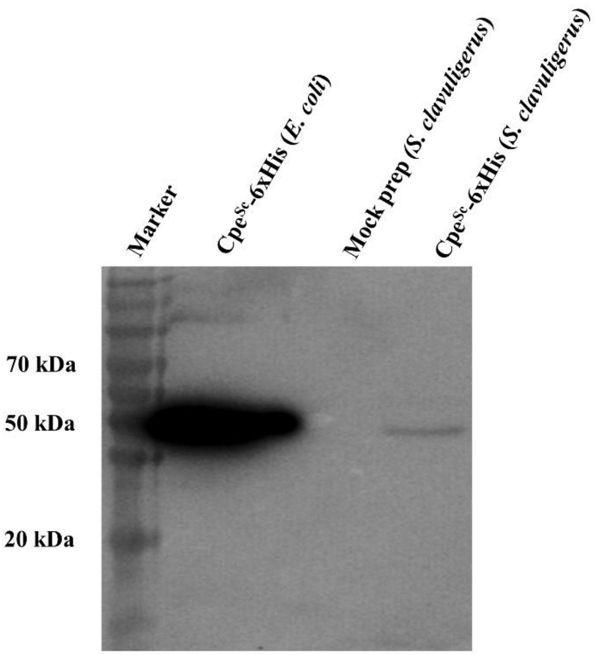

S1 Fig. Western blot analysis of C-terminal 6×His tagged Cpe (Cpe$^{Sc\text{-}6\times His}$) expressed and purified from *E. coli* (using pET 30b-*cpe*^{Sc}) or expressed in *Streptomyces clavuligerus* (using pHM:*cpe*$^{Sc\text{-}6\times his}$). Purified protein (*E. coli*) or cell free lysates (*S. clavuligerus*) were used in the analysis along with anti-6×His antibodies for detecting epitope-tagged Cpe. The lane labeled as "Mock prep" contains *S. clavuligerus* pHM11a empty vector lysate as control to account for any non-specific antibody binding. The size of the band corresponding to Cpe$^{Sc\text{-}6\times His}$ in all lanes was approximately 50-55 kDa, and the prestained protein ladder (Marker) was used as a reference for estimating molecular weights during 12% SDS-PAGE.
