# Supplementary material for: In vivo functional analysis of a class A β-lactamase-related protein essential for clavulanic acid biosynthesis in Streptomyces clavuligerus
Source: PLoS One. 2019 Apr 23;14(4):e0215960. doi: 10.1371/journal.pone.0215960 (PMC6478378; doi:10.1371/journal.pone.0215960)
Supplement: S2 Fig — The peak corresponding to imidazole-derivatized clavulanic acid (CA) is indicated and was only observed when the full-length protein was used in the analysis. (PDF) [file pone.0215960.s002.pdf]

S2 Fig.

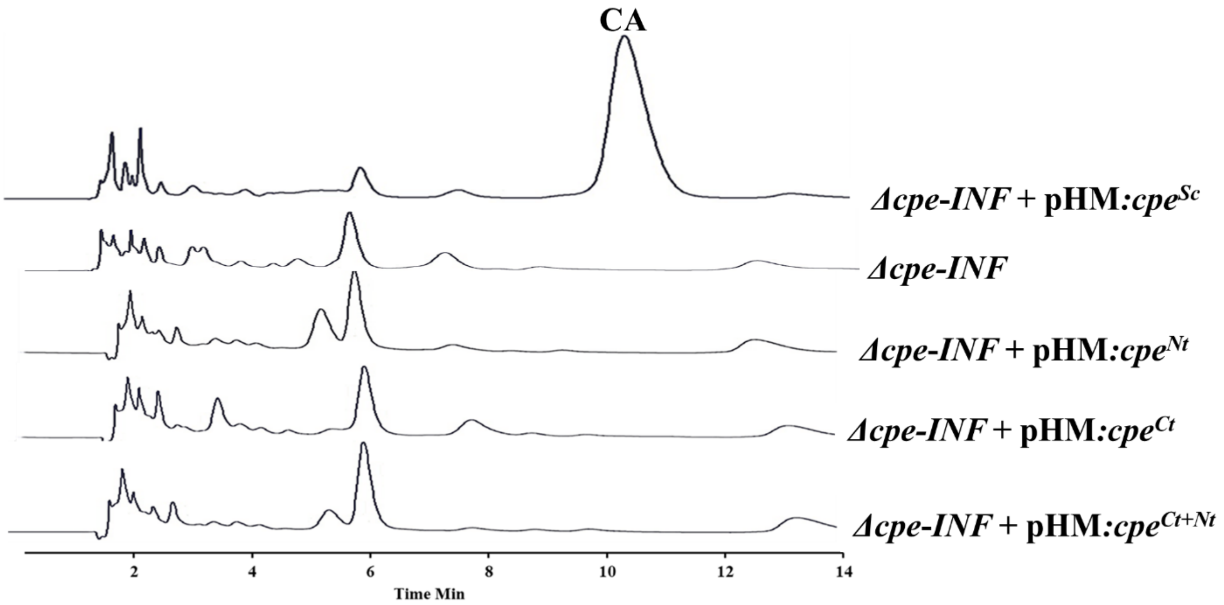

S2 Fig. HPLC analysis of 96 hour SA culture supernatants from the *S. clavuligerus Δcpe-INF* strain expressing full-length Cpe^{Sc} (pHM:cpe^{Sc}), its N-terminus (pHM:cpe^{Nt}), C-terminus (pHM:cpe^{Ct}) or both the N- and C-terminal domains at the same time as separate peptides (pHM:cpe^{Ct+Nt}). The peak corresponding to imidazole-derivatized clavulanic acid (CA) is indicated and was only observed when the full length protein was used in the analysis.
