# Supplementary material for: In vivo functional analysis of a class A β-lactamase-related protein essential for clavulanic acid biosynthesis in Streptomyces clavuligerus
Source: PLoS One. 2019 Apr 23;14(4):e0215960. doi: 10.1371/journal.pone.0215960 (PMC6478378; doi:10.1371/journal.pone.0215960)
Supplement: S3 Fig — The complete gene sequences starting from initiation (ATG) to the stop (TGA) codon for each gene were determined as part of the current study and are reported. (PDF) [file pone.0215960.s003.pdf]

**S3 Fig.**

***>Streptomyces jumonjinensis* (*cpe$^{sj}$*)**

ATGATCGAAGCTGCCGGTTCCGTCCCCAGCGCCGCCCAGGCCGCGCTCGCCGCCGACGGCTCCCCGATGGGCGAGGCGGCCC
GGTGGGCGGTGAATTTACTCACGGCGCCCGACATCGCCTCTGACGAGGAAATGGCCGCACGCTTCATGCCCACCTTCGGGGCC
GGAGTCGCGGGCAAGTTCTCCCCCACTCTTCGCGAGTGGCGGGCGCAGGGCCCCTACACCGTCGAGGACTACCAGGCGGTCG
CCCACAAGAGCTGGGTCACGCTGACGGGGCCTGCGGGCGAACGCCACACCCTGTCGCTGACGCTCGACTCCAACGGGGTGAT
CCGCATCCTCACCCTCCGGCCCGAAATGGCGATCCCGCAGACGCGGACCTGGGACGAGCTGGAGGAGGCGCTGCGCATACCC
GGCATCGAGCACTCCGTGCTCGCCGCCCGGCTCACCCCCTCGGGGCCCGTCGTCCTGCATGAGACGGACGCCGACCGGCCGAT
GGCCACCGGGTCGGCGAACAAGCTCTATGTGATGCGCGCGCTCATCGAGGCGCTGGAGAGCGGGAAGATCAGCTGGGACGA
CGAGGTCACCGTCACACCGGAGCTGCGCAGTCTGCCCACGGGCGATATGCAGGACCTCCCCGACGGGACCCGGGTCTCGGTG
CGCGAGACGGCGCACAAGATGATCGTGCTGAGCGACAACACGGCCGCCGACATCTTCGTGGACCGGCTGGGCCGGGACGCC
GTGGAGCGCGCCGTGGCGGCCTCCGGGCACCATGACCCGGCCCTGATGCGTCCGTTCCTGAGCAGCCAGGAGTTCTTCGAAC
TCGGCTGGGGCGCGCGGGAGAGGCGTGCCGAGTGGACGCGGCGGGACGAGGCCGGGCGGCGCGAGATGCTGCGCGGGAT
CACCGGGCCGATGACCGTGCGCGGCTCCGACCTGGGCGAGACGGTGCACCAGCTGGGCATCGACTGGATGATGAACGCCTAC
GACGTGCTCCATGTCCTGGCGGGGCTCATGGAGGACAGTGAGCGGGATACCACCGGCGCCGTCGAGCGGATCCTGACCGCCT
ATCCGGGCATCGTCGCCGACCCCGCCATCTGGGGCAGGGTGTACTTCAAGGCCGGCTCGTCCCCCGGCGTGATGATGTTCTGC
TGGCTGCTCCAGAACCGGGCCGGCGAAACGTATGTCCTGGTGCTGCGGCAGATGGCCGATGAGCAGAAGCCCATCGGCGAC
GGCCTGTATCTGCGCGGAGTCGGCGCCCGTGTCATCGAGTCCGGCCTGCTGGAGTCCGGCGCGGCCGTCGGCGCGCGCGCCA
GACAGGCGGACGGGGTCAGCACCCCATGA

***>Streptomyces katsurahamanus* (*cpe$^{sk}$*)**

ATGATCGAAGCTGCCGGTTCCGTCCCCAGCGCCGCCCAGGCCGCGCTCGCCGCCGACGGCTCCCCGATGGGCGAGGCGGCCC
GGTGGGCGGTGAATTTACTCACGGCGCCCGACATCGCCTCTGACGAGGAAATGGCCGCACGCTTCATGCCCACCTTCGGGGCC
GGAGTCGCGGGCAAGTTCTCCCCCACTCTTCGCGAGTGGCGGGCACAGGGCCCCTACACCGTCGAGGACTACCAGGCGGTCG
CCCACAAGGGCTGGGTCACGCTGACGGGACCGGCGGGCGAACGCCACACCCTGTCGCTGACGCTCGACTCCAACGGGGTGAT
CCGCATCCTCACCCTCCGGCCCGAGATGGCGATCCCCCAGACGCGGACCTGGGACGAGCTGGAGGAGGCGCTGCGCATACCC
GGCATCGAGCACTCCGTGCTCGCCGCCCGGCTCACCCCCTCGGGGCCCGTCGTCCTGCATGAGACGGACGCCGACCGGCCGAT
GGCCACCGGGTCGGCGAACAAGCTCTATGTGATGCGCGCGCTCATCGAGGCGCTGGAGAGCGGGAAGATCAGCTGGGACGA
CGAGGTCACCGTCACACCGGAGCTGCGCAGTCTGCCCACGGGCGATATGCAGGACCTCCCCGACGGGACCCGGGTCTCGGTG
CGCGAGACGGCGCACAAGATGATCGTGCTGAGCGACAACACGGCCGCCGACATCTTCGTGGACCGGCTGGGCCGGGACGCC
GTGGAGCGCGCCGTGGCGGCCTCCGGGCACCATGACCCGGCCCTGATGCGTCCGTTCCTGAGCAGCCAGGAGTTCTTCGAAC
TCGGCTGGGGCGCGCGGGAGCGGCGCGCCGAGTGGACGCGGCGGGACGAGGCCGGGCGGCGCGAGATGCTGCGCGGGAT
CACCGGGCCGATGGCCGTGCGCGGCTCCGACCTGGGCGAGACGGTGCACCAGCTGGGCATCGACTGGATGATGAACGCCTAC
GACGTGCTCCATGTCCTGGCGGGGCTCATGGAGGACAGCGAGCGGGATACCACCGGCGCCGTCGAGCGGATCCTGACCGCCT
ATCCGGGCATCGTCGCCGACCCCGCCATCTGGGGCAGGGTGTACTTCAAGGCCGGCTCGTCCCCCGGCGTGATGATGTTCTGC
TGGCTGCTCCAGAACCGGGCCGGCGAAACGTACGTCCTGGTGCTGCGACAGATGGCCGACGAGCAGAAGCCCATCGGCGAC
GGCCTGTATCTGCGCGGAGTCGGCGCCCGCGTCATCGAGTCCGGCCTGCTGGAGTCCGGCGCGGCCGTCGGCGCGCGCGCCA
GACAGGCGGACGGGGTCAGCACCCCATGA

**S3 Fig.** DNA sequences of *cpe* homologues from *S. jumonjinensis and S. katsurahamanus.* The complete gene sequences starting from initiation (ATG) to the stop (TGA) codon for each gene were determined as part of the current study and are reported.
