# Supplementary material for: In vivo functional analysis of a class A β-lactamase-related protein essential for clavulanic acid biosynthesis in Streptomyces clavuligerus
Source: PLoS One. 2019 Apr 23;14(4):e0215960. doi: 10.1371/journal.pone.0215960 (PMC6478378; doi:10.1371/journal.pone.0215960)
Supplement: S4 Fig — Analysis was performed with Clustal Omega (ver 1.2.1) using translated Cpe amino acid sequences from S. clavuligerus (WP_003952519.1), S. flavogrisius (WP_014152684.1), S. viridis (WP_015787620), S. jumonjinensis and S. katsurahamanus. The DNA sequences of cpe from the latter two producers (S. jumonjinensis and S. katsurahamanus) were determined as part of the current study and are reported in S3 Fig. The boxes in black highlight the conserved SXXK, SDN and KTG/KAG motifs present in class A β-lactamses and the respective Cpe proteins, whereas the box in blue represents the N-terminus domain indentifed in CpeSc. The arrows indicate amino acids from CpeSc that were selected for mutagenesis and the ones highlighted in red were shown to be essential for in vivo clavulanic acid production in S. clavuligerus. (PDF) [file pone.0215960.s004.pdf]

S4 Fig.

```
                                                    Ser27
S.viridis          MLTTTET---------SGADATTARRPLVESRMRWAVEMLAEPGPGLAEDPRDHFVAAFV
S.flavogriseus     MIDLPGS-----DASARARAALDGDDTPLGSRTRWAAELLTAPEAPRDDDLSEHFVPVFL
S.clavuligerus     MMKKADSVPTPAEAALAAQTALAADDSPMGDAARWAMGLLTSSGLPRPEDVAARFIPTFA
S.jumonjinensis    MIEAAGSVPS------AAQAALAADGSPMGEAARWAVNLLTAPDIASDEEMAARFMPTFG
S.katsurahamanus   MIEAAGSVPS------AAQAALAADGSPMGEAARWAVNLLTAPDIASDEEMAARFMPTFG
                   *:    :          .  *  .     :  .  ***  :*  :        ::   :*:  .*

                                             Lys89 Trp91                     Arg115
S.viridis          DKHGDGLSALLDSWRAQGPFTVESYTPVAHKGWATLIGPTGDRFTLSIVIDSSGLIRGLG
S.flavogriseus     ARHPQGFAATLEQWRGLGPFTVTEYQPVAHKSWVTLEGPSGTRSTLSLTLDSTGLIRVLH
S.clavuligerus     A--AGNFAETVREWRSKGPFTVRAYHPVAHKGWVVLSAPAGVRYILSLTLDSSGLIRILT
S.jumonjinensis    AGVAGKFSPTLREWRAQGPYTVEDYQAVAHKSWVTLTGPAGERHTLSLTLDSNGVIRILT
S.katsurahamanus   AGVAGKFSPTLREWRAQGPYTVEDYQAVAHKGWVTLTGPAGERHTLSLTLDSNGVIRILT
                         :: :  .**. **:**  *  ****.*..* .*:* *  **:.:**.*:** *

                                                                      Ser173 Lys176
S.viridis          LGPELVIPPLRDWDELDAALDVPGVTSTALVARLVDDRWVSLYERSPDRLMPTGSAYKLY
S.flavogriseus     LGPEVVTPAVHGWDDLEAALEQPGVTWSALVTREEGGTTETLYAREADRPMPNGSSYKLY
S.clavuligerus     LKPETVIPDMVTWNDVEETLHTPGVQHSVYAVRLTPDGHEVLHASAPERPMPTGSAYKLY
S.jumonjinensis    LRPEMAIPQTRTWDELEEALRIPGIEHSVLAARLTPSGPVVLHETDADRPMATGSANKLY
S.katsurahamanus   LRPEMAIPQTRTWDELEEALRIPGIEHSVLAARLTPSGPVVLHETDADRPMATGSANKLY
                   * **  . *    *:::: :* **:  :. ..*   .    *:    :* * .**: ***

                                            Ser206                        Ser234
S.viridis          LLRALARAIEAGTVRWDEELTLLPQLRSLPTGEMQDLPDGARATVREVAHKMIAMSDNTA
S.flavogriseus     LMRALADAVASGSVRWDDEVTVTPALRSLPTGDMQELPDGARVSVRETAYKMIAMSDNTA
S.clavuligerus     LMRALVAEIEKGTVGWDEILTLTPELRSLPTGDMQDLPDGTRVTVRETAHKMIALSDNTG
S.jumonjinensis    VMRALIEALESGKISWDDEVTVTPELRSLPTGDMQDLPDGTRVSVRETAHKMIVLSDNTA
S.katsurahamanus   VMRALIEALESGKISWDDEVTVTPELRSLPTGDMQDLPDGTRVSVRETAHKMIVLSDNTA
                   ::***    :  *.: **: :*: * *******:**:****:*.:***.*:***.:****.

S.viridis          ADMVLHRLGRDAVHRAVVDSGHQDPDVLRPFLSSRELFEIGWGDDPSLLPEWSAGDRAQR
S.flavogriseus     ADLIQDLLGRDAVERAVVEGGHSDPALLRPFLSSHEVFEIGWGA-PGLRAEWVAADEDGR
S.clavuligerus     ADLVADRLGREVVERSLAAAGHHDPSLMRPFLTSHEVFELGWGD-PERRAEWVRQDEAGR
S.jumonjinensis    ADIFVDRLGRDAVERAVAASGHHDPALMRPFLSSQEFFELGWGA-RERRAEWTRRDEAGR
S.katsurahamanus   ADIFVDRLGRDAVERAVAASGHHDPALMRPFLSSQEFFELGWGA-RERRAEWTRRDEAGR
                   **:. .  ***:.*.*::. .** ** ::****:*:*.**:***        **    *.  *

                                 Arg311     Gln321  Trp326             Arg346
S.viridis          YELLRRIERPLTTRMRDLNRPVYHLGLDWFLSARDVANALAGVWRDTTRDRTGTIREIVT
S.flavogriseus     RRLLDRIAGPMTVRGSDLGATVHQLGLDWHMSAWDVARVLGALADDGTRDTSGAVEDILT
S.clavuligerus     RELLEKMAGVMTVRGSDLGATVHQLGIDWHMDAFDVVRVLEGLLQDSGRDTSGTVEEILT
S.jumonjinensis    REMLRGITGPMTVRGSDLGETVHQLGIDWMMNAYDVLHVLAGLMEDSERDTTGAVERILT
S.katsurahamanus   REMLRGITGPMAVRGSDLGETVHQLGIDWMMNAYDVLHVLAGLMEDSERDTTGAVERILT
                    .:*  :   ::.*  **.  *::**:** :.* ** ..* .:  *  ** :*::. *:*

          Tyr359               Lys375 Ser378
S.viridis          TYPGVPIDGERWPVAVFKGGSSPGVVMFCWLLVDPAGVPHVVVLQQCGEDPGQVGDGLRL
S.flavogriseus     AYPGVPVDAARWPRVVFKGGSCPGVMMFCWLLQDPDGVRYTLVLQQRADDQKLIGDGQFL
S.clavuligerus     AYPGLLIDEERWRRVYFKAGSSPGVMMFCWLLQDHAGISYVLVLRQSADEQRLIGDGLFL
S.jumonjinensis    AYPGIVADPAIWGRVYFKAGSSPGVMMFCWLLQNRAGETYVLVLRQMADEQKPIGDGLYL
S.katsurahamanus   AYPGIVADPAIWGRVYFKAGSSPGVMMFCWLLQNRAGETYVLVLRQMADEQKPIGDGLYL
                   :***:  *   *  . **.**.***:****** :  *  :.:**:* .::   :***  *

                   Arg418
S.viridis          RGLGDRIIHSLLP----------------------------
S.flavogriseus     RGMGGRVIGSG------------------------------
S.clavuligerus     RGIGAKIIEAEAKLLSSGERRGAGTAAAGDDRASAGEAARR
S.jumonjinensis    RGVGARVIESG--LLESGAAVGARARQADGVSTP-------
S.katsurahamanus   RGVGARVIESG--LLESGAAVGARARQADGVSTP-------
                   **:* ::* :
```

S4 Fig. Multiple sequence alignment of different Cpe proteins described in the current study. Analysis was performed with Clustal Omega (ver 1.2.1) using translated Cpe amino acid sequences from *S. clavuligerus* (WP_003952519.1), *S. flavogrisius* (WP_014152684.1), *S. viridis* (WP_015787620), *S. jumonjinensis* and *S. katsurahamanus.* The DNA sequences of *cpe* from the latter two producers (*S. jumonjinensis* and *S. katsurahamanus*) were determined as part of the current study and are reported in S3 Fig. The boxes in black highlight the conserved SXXK, SDN and KTG/KAG motifs present in class A β-lactamses and the respective Cpe proteins, whereas the box in blue represents the N-terminus domain indentifed in Cpe^{Sc}. The arrows indicate amino acids from Cpe^{Sc} that were selected for mutagenesis and the ones highlighted in red were shown to be essential for *in vivo* clavulanic acid production in *S. clavuligerus*.
