# Supplementary material for: In vivo functional analysis of a class A β-lactamase-related protein essential for clavulanic acid biosynthesis in Streptomyces clavuligerus
Source: PLoS One. 2019 Apr 23;14(4):e0215960. doi: 10.1371/journal.pone.0215960 (PMC6478378; doi:10.1371/journal.pone.0215960)
Supplement: S5 Fig — Multiple sequence alignments using the predicted amino acid sequences of Cpe proteins listed in S4 Fig. and class A β-lactamases including Bla (from the S. clavuligerus cephamycin C biosynthetic gene cluster, CAA90895.1) and TEM-1 (from E.coli, AMM70781.1) were used to prepare the tree, and bootstrap analyses were performed using 100 replicates. All positions containing gaps were eliminated during the analysis and the number next to each node represents the percentage of trees in which the respective topologies were observed. (PDF) [file pone.0215960.s005.pdf]

S5 Fig.

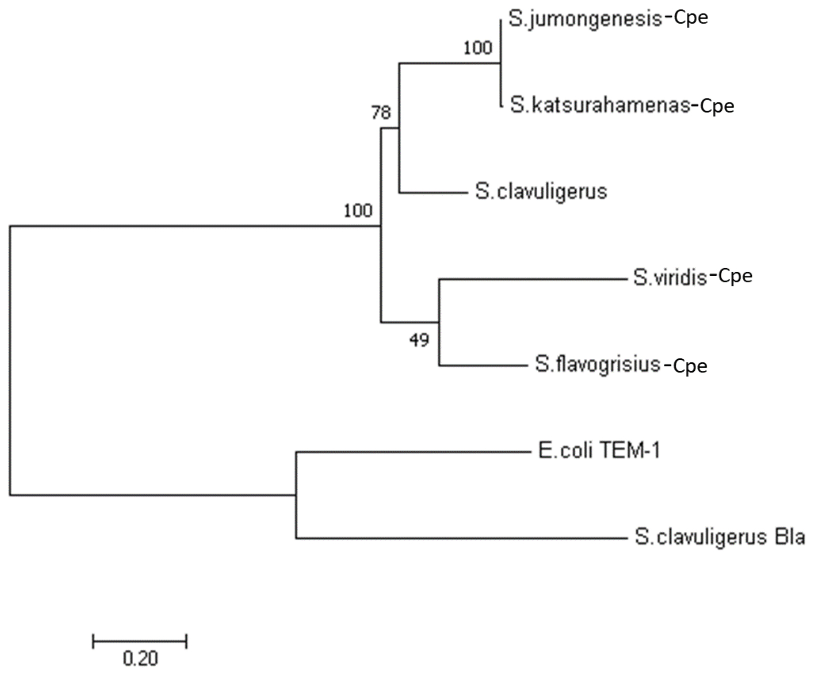

S5 Fig. Phylogenetic relationship between select class A β-lactamases and Cpe proteins described in the current study. Multiple sequence alignments using the predicted amino acid sequences of Cpe proteins listed in S4 Fig. and class A β-lactamases including Bla (from the *S. clavuligerus* cephamycin C biosynthetic gene cluster, CAA90895.1) and TEM-1 (from *E.coli*, AMM70781.1) were used to prepare the tree, and bootstrap analyses were performed using 100 replicates. All positions containing gaps were eliminated during the analysis and the number next to each node represents the percentage of trees in which the respective topologies were observed.
