# Supplementary material for: In vivo functional analysis of a class A β-lactamase-related protein essential for clavulanic acid biosynthesis in Streptomyces clavuligerus
Source: PLoS One. 2019 Apr 23;14(4):e0215960. doi: 10.1371/journal.pone.0215960 (PMC6478378; doi:10.1371/journal.pone.0215960)
Supplement: S6 Fig — Clavulanic acid (CA) production was monitored at 311nm following imidazole derivatization. (PDF) [file pone.0215960.s006.pdf]

S6 Fig.

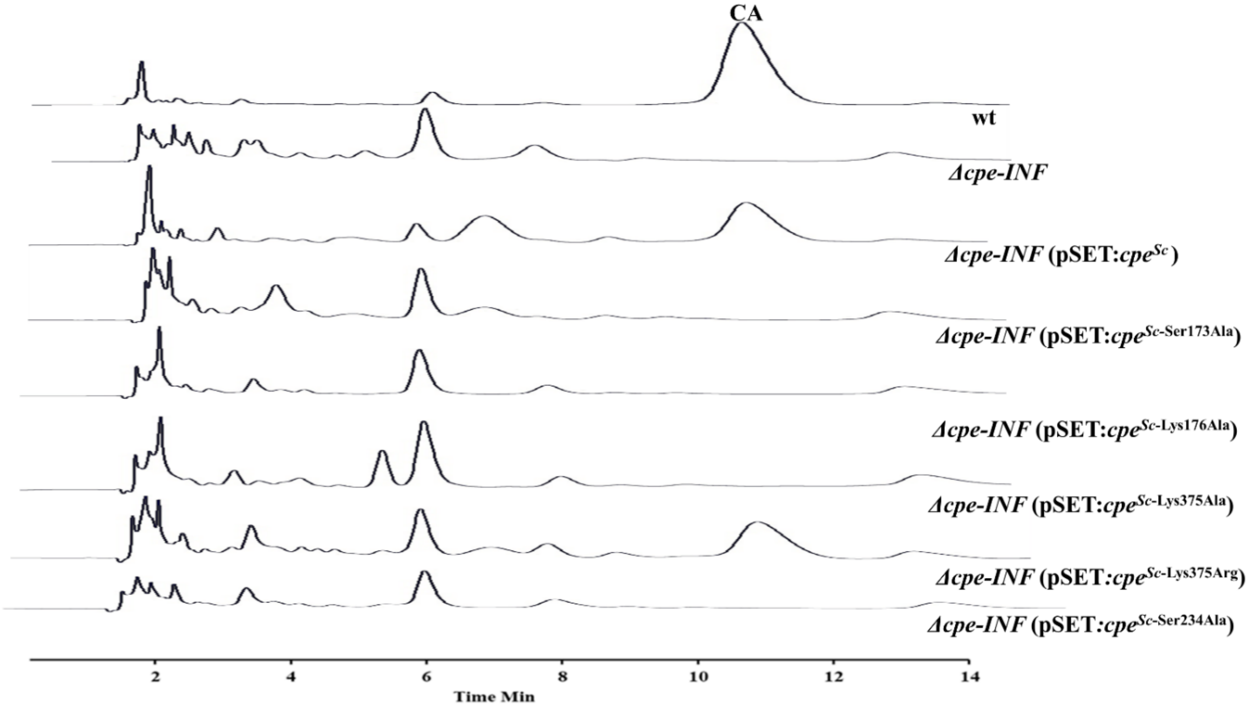

S6 Fig. HPLC analysis of 96 hour *S. clavuligerus* SA culture supernatants from the wt strain for comparison with the *Δcpe-INF* mutant expressing Cpe^{Sc} or select single amino acid variants (Ser_{173}Ala, Lys_{176}Ala, Lys_{375}Ala, Lys_{375}Arg Ser_{234}Ala) of the protein *in trans* using plasmid pHM11a. Clavulanic acid (CA) production was monitored at 311nm following imidazole derivatization.
