# Supplementary material for: In vivo functional analysis of a class A β-lactamase-related protein essential for clavulanic acid biosynthesis in Streptomyces clavuligerus
Source: PLoS One. 2019 Apr 23;14(4):e0215960. doi: 10.1371/journal.pone.0215960 (PMC6478378; doi:10.1371/journal.pone.0215960)
Supplement: S1 Table — (PDF) [file pone.0215960.s007.pdf]

S1 Table. Oligonucleotide primers used for cloning and sequencing in the current study.

Primer Name	Primer Sequence 5′→3′	Description
cpe-RD-F/R	**(F)** ATG ATG AAG AAA GCT GAT TCC GTC CCG ACC CCG GCT GAG ATT CCG GGG ATC CGT CGA CC<br>**(R)** TCA TCG CCG GGC GGC TTC TCC GGC GCT CGC GCG GTC GTC TGT AGG CTG GAG CTG CTT C	Forward (F) and reverse (R) primers for introduction of *apra-oriT* cassette in place of *cpe* in cosmid 12B8 (ReDirect)
cpe-CONF-F/R	**(F)** CCG ACA AGG AGC GAT GAT GA<br>**(R)** GGA TCA CAC CGA AGC CCA GT	Forward (F) and reverse (R) primers for confirming deletion of *cpe* in cosmid 12B8
pIJ773-R	TCG CTA TAA TGA CCC CGA AG	Sequencing primer used for confirming insertion of *apra-oriT* cassette in place of *cpe*
pWE15-amp-RD-F/R	**(F)** TGA TAA ATG CTT CAA TAA TAT TGA AAA AGG AAG AGT ATG ATT CCG GGG ATC CGT CGA CC<br>**(R)** AATCTAAAG TAT ATA TGA GTA AAC TTG GTC TGA CAG TTA TGT AGG CTG GAG CTG CTT C	Forward (F) and reverse (R) primers for introduction of *hyg-oriT* cassette in place of *amp* in cosmid 12B8 (ReDirect)
pIJ799-F/R	**(F)** CCCTGATAATGCTTCAATAATATTGAAAA<br>**(R)** AAT CAA TCT AAA GTA TAT ATG AGT AAA CTT GGT C	Forward (F) and reverse (R) primers for confirming replacement of *amp* in cosmid 12B8
HygR-F/R	**(F)** CGC ATA GAC GTC GGT GAA GT<br>**(R)** TAC CTG GTG ATG AGC CGG AT	Forward (F) and reverse (R) primers for confirming presence/absence of *hyg* in *cpe* mutants
blip-FLAG-F/R	**(F)** ATA CAT ATG AGG ACA GTG GGG ATC G<br>**(R)** ATG GAT CCT CAC TTG TCG TCG TCG TCC TTG TAG TCT ACA AGG TCC CAC TGC CGC TTG	Forward (F) and reverse (R) primers for amplification of *blip* from *S. clavuligerus* with C-terminal FLAG tag
ccaR-FLAG-F/R	**(F)** TAC ATA TGA ACA CCT GGA ATG ATG TGA C<br>**(R)** ATG GAT CCT CAC TTG TCG TCG TCG TCC TTG TAG TCG GCC GGG GTA CCG ACC	Forward (F) and reverse (R) primers for amplification of *ccaR* from *S. clavuligerus* with C-terminal FLAG tag
cpe-FLAG-F/R	**(F)** TAC ATA TGA TGA AGA AAG CTG ATT CCG TC<br>**(R)** ATG GAT CCT CAC TTG TCG TCG TCG TCC TTG TAG TCT CGC CGG GCG GCT TC	Forward (F) and reverse (R) primers for amplification of *cpe* from *S. clavuligerus* with C-terminal FLAG tag
cpe-SC-F/R	**(F)** TAATA CAT ATG ATG AAG AAA GCT GAT TCC G<br>**(R)** TGATA GGA TCC TCA TCG CCG GGC GGC TTC	Forward (F) and reverse (R) primers for amplification of *cpe* from *S. clavuligerus*
cpe-SF-F/R	**(F)** ATTACATATGA TCG ATC TAC CCG GTT CCG<br>**(R)** TAATAAGCTTC ACCGGG AGG TGC CGG	Forward (F) and reverse (R) primers for amplification of *cpe* from *S. flavogriseus*
cpe-SV-F/R	**(F)** TATACATATGT TGA CCA CTA CCG AGA C<br>**(R)** ACATAAGCTTC ACG GCA GCA ACG AGT G	Forward (F) and reverse (R) primers for amplification of *cpe* from *S. viridis*

S1 Table (Continued)		
cpe-SK-F/R	**(F)** ACTAGCATATG ATGATSRADVHWSCYGRT<br>**(R)** TGCAA AAGCTT TCAWSDYSGGRNYSSHKN	Forward (F) and reverse (R) degenerate primers for amplification and cloning of *cpe* from *S. katsurahamanus*
cpe-SJ-F/R	**(F)** ACTAG CATATG ATGATSRADVHWSCYGRT<br>**(R)** TGCAA AAGCTT TCAWSDYSGGRNYSSHKN	Forward (F) and reverse (R) degenerate primers for amplification and cloning of *cpe* from *S. jumonjinensis*
cpe^{Ct}-F/R	**(F)** ATATCATATGATGGTCACGTGGAACGATGTC<br>**(R)** ATATGGATCCTCATCGCCGGGCGGCTTC	Forward (F) and reverse (R) primers for amplification of the C-terminal domain of Cpe
cpe^{Nt}-F/R	**(F)** ATAATCATATGATGATGAAGAAAGCTGATTC<br>**(R)** ATATAGGATCCTCAGTCGGGTATGACGGTCTC	Forward (F) and reverse (R) primers for amplification of the N-terminal domain of Cpe
