# Supplementary material for: In vivo functional analysis of a class A β-lactamase-related protein essential for clavulanic acid biosynthesis in Streptomyces clavuligerus
Source: PLoS One. 2019 Apr 23;14(4):e0215960. doi: 10.1371/journal.pone.0215960 (PMC6478378; doi:10.1371/journal.pone.0215960)
Supplement: S2 Table — (PDF) [file pone.0215960.s008.pdf]

S2 Table. Gene specific primer pairs used for RT-PCR analysis in the current study.

Gene	Primer Function: Sequence (5′→3′)
ceaS2	Forward: ATCGACTTCGTTCTGACCCG<br>Reverse: GGTGTCGTTCGGGAAGATGT
cas2	Forward: GCAAGCGGCTGGTGATGG<br>Reverse: GGTCTCCGAGGACAGGTAGTGC
oat2	Forward: CACCGTCCTCGCCTCCAC<br>Reverse: CGTTCTCCTCGCCCTCCAG
oppA1	Forward: CGGGGTACGGGGAGTGG<br>Reverse: CGGAGGAAGTTCCAGGTGTA
claR	Forward: CGGGCGGCGGTTCTT<br>Reverse: TCGTCGAGCAGGGGTTCC
cyp	Forward: ACGAACTCGACGGCTATCTG<br>Reverse: ACATCGGGACCATCTCCTC
car	Forward: GTCTACCAGGCCACGAAGTT<br>Reverse: GATCCGCTGCTCGTACATCT
cpe (*orf12*)	Forward: GGCGATGGGGCTGCTGAC<br>Reverse: GTGCGCGACGGGGTGGTA
orf13	Forward: CTGCGCTGGCTGCTGGTGTA<br>Reverse: CTGCCGCCGGGAGATGC
orf14	Forward: CGAACGACGACGAAACG<br>Reverse: CAGCGAGCCGACCATGT
oppA2	Forward: CCCACCTATCTCATCCCGC<br>Reverse: CATCAGATGGTCGAAGTCGGA
orf16	Forward: TTCCTGGCCGACATGACCAA<br>Reverse: CCGTACTTGCGCAGCAGATT
gcas	Forward: GGTCAACTGGAGCCTGTGTA<br>Reverse: CCGCGAACTTGGCATAGTC
*pbpA*1	Forward: CAAGTACCAGCGCACCTACA<br>Reverse: CGCTCAATACGCTGTCGAAC
hrdB	Forward: CGCGGCATGCTCTTCCT<br>Reverse: AGGTGGCGTACGTGGAGAAC
