# Supplementary material for: In vivo functional analysis of a class A β-lactamase-related protein essential for clavulanic acid biosynthesis in Streptomyces clavuligerus
Source: PLoS One. 2019 Apr 23;14(4):e0215960. doi: 10.1371/journal.pone.0215960 (PMC6478378; doi:10.1371/journal.pone.0215960)
Supplement: S3 Table — (PDF) [file pone.0215960.s009.pdf]

S3 Table. Primer pairs used for site directed mutagenesis of *cpe*^{Sc}.

Primer	Primer Sequence 5′→3′	Forward/Reverse Primer
Cpe-Ser27Ala-F	GCCCATGGGGGCGTCGTCGGCCG	Forward
Cpe-Ser27Ala-R	CGGCCGACGACGCCCCCATGGGC	Reverse
Cpe-Ser173Ala-F	GCTTGTACGCCGCCCCGGTCGGCAT	Forward
Cpe-Ser173Ala-R	ATGCCGACCGGGGCGGCGTACAAGC	Reverse
Cpe- Tyr359Ala-F	CAGCAGACCGGGGGCGGCGGTGAGGATC	Forward
Cpe- Tyr359Ala-R	GATCCTCACCGCCGCCCCCGGTCTGCTG	Reverse
Cpe-Ser206Ala-F	CCCGTGGGCAGGGCGCGCAGCTCGGG	Forward
Cpe-Ser206Ala-R	CCCGAGCTGCGCGCCCTGCCCACGGG	Reverse
Cpe-Ser234Ala-F	CGCCGGTGTTGTCGGCCAGCGCGATCATCT	Forward
Cpe-Ser234Ala-R	AGATGATCGCGCTGGCCGACAACACCGGCG	Reverse
Cpe-Lys375Ala-F	GCTGGAACCGGCCGCGAAGTAGACCCGGC	Forward
Cpe-Lys375Ala-R	GCCGGGTCTACTTCGCGGCCGGTTCCAGC	Reverse
Cpe-Ser378Ala-F	ACCGGGGCTGGCACCGGCCTTGAAG	Forward
Cpe-Ser378Ala-R	CTTCAAGGCCGGTGCCAGCCCCGGT	Reverse
Cpe-Lys89Ala-F	CACGACCCAGCCCGCGTGCGCGACGGGG	Forward
Cpe-Lys89Ala-R	CCCCGTCGCGCACGCGGGCTGGGTCGTG	Reverse
Cpe-Trp91Ala-F	CGACAGCACGACCGCGCCCTTGTGCGCG	Forward
Cpe-Trp91Ala-R	CGCGCACAAGGGCGCGGTCGTGCTGTCG	Reverse
Cpe-Lys375Arg-F	GAACCGGCCCTGAAGTAGACCCGGCGC	Forward
Cpe-Lys375ArgR	GCGCCGGGTCTACTTCAGGGCCGGTTC	Reverse
Cpe-Arg418Lys-F	CGGTCTGTTCCTGGCCGGGATCGGCGCC	Forward
Cpe-Arg418Lys-R	GGCGCCGATCCCGGCCAGGAACAGACCG	Reverse
Cpe-Arg311Ala-F	CAGGTCGGAGCCGGCGACGGTCATCACC	Forward
Cpe-Arg311Ala-R	GGTGATGACCGTCGCCGGCTCCGACCTG	Reverse
Cpe-Glu321Ala-F	GATGCCGAGCGCATGGACCGTCGCGCCC	Forward

S3 Table (Continued)

Cpe-Glu321Ala-R	GGGCGCGACGGTCCATGCGCTCGGCATC	Reverse
Cpe-Trp326Ala-F	GCTCGGCATCGACGCGCACATGGACGCC	Forward
Cpe-Trp326Ala-R	GGCGTCCATGTGCGCGTCGATGCCGAGC	Reverse
Cpe-Arg346Ala-F	CCGCTGGTGTCCGCTCCGCTGTCCTGGA	Forward
Cpe-Arg346Ala-R	TCCAGGACAGCGGAGCGGACACCAGCGG	Reverse
Cpe-Lys176Ala-F	CGCATCAGATAGAGCGCGTACGCCGACCCGGT	Forward
Cpe-Lys176Ala-R	ACCGGGTCGGCGTACGCGCTCTATCTGATGCG	Reverse
Cpe-Arg115Ala-F	CAGGGTGAGGATGGCGATCAGCCCGCTG	Forward
Cpe-Arg115Ala-R	CAGCGGGCTGATCGCCATCCTCACCCTG	Reverse
