# Supplementary material for: In vivo functional analysis of a class A β-lactamase-related protein essential for clavulanic acid biosynthesis in Streptomyces clavuligerus
Source: PLoS One. 2019 Apr 23;14(4):e0215960. doi: 10.1371/journal.pone.0215960 (PMC6478378; doi:10.1371/journal.pone.0215960)
Supplement: S4 Table — (PDF) [file pone.0215960.s010.pdf]

S4 Table. LC-MS analysis of SA culture supernatants from wt *S. clavuligerus* and *Δcpe-INF* mutant strains for detecting clavulanic acid and pathway intermediates.

**Strain (plasmid)**[a]	**Metabolite**[b,c]						
	**Clavaminic acid** (*m/z*=223/155)	***N*- acetyl clavaminic acid** (*m/z*=265/197)	***N*-glycyl clavaminic acid** (*m/z*=280/212)	***N*- acetyl glycyl clavaminic acid** (*m/z*=322/254)	**C-glycyl clavaminic acid** (*m/z*=324/256)	**Clavaldehyde** (*m/z*=222/154)	**Clavulanic acid** (*m/z*=224/156)
**wt**	-	-	-	-	-	-	+
**Δ*cpe-INF***	-	-	-	-	-	-	-
Δcpe-INF **(pSET:*cpe*^{Sc})**	-	-	-	-	-	-	+
Δcpe-INF **(p*SET:cpe*$^{Sc\text{-Trp91Ala}}$)**	-	-	-	-	-	-	+
Δcpe-INF **(pSET:*cpe*$^{Sc\text{-Ser173Ala}}$)**	-	-	-	-	-	-	-
Δcpe-INF **(pSET:*cpe*$^{Sc\text{-Lys176Ala}}$)**	-	-	-	-	-	-	-
Δcpe-INF **(p*SET:cpe*$^{Sc\text{-Ser234Ala}}$)**	-	-	-	-	-	-	-
Δcpe-INF **(p*SET:cpe*$^{Sc\text{-Gln321Ala}}$)**	-	-	-	-	-	-	+
Δcpe-INF **(pSET:*cpe*$^{Sc\text{-Tyr359Ala}}$)**	-	-	-	-	-	-	-
Δcpe-INF **(pSET:*cpe*$^{Sc\text{-Lys375Ala}}$)**	-	-	-	-	-	-	-
***Δcpe-INF*(pHM:*cpe*^{Ct})**	-	-	-	-	-	-	-

[a] Select strains were fermented for 96 hours and culture supernatants were subjected to LC-MS analysis after imidazole derivatization.

[b] Metabolites and respective *m/z* values of corresponding imidazole derivatives/fragmentation products/ions are indicated. Some clavaminic acid derived 5*S* metabolites reported to accumulate in certain *S. clavuligerus* mutant strains were also included in the analysis.

[c] (+) and (-) indicates that ions corresponding the metabolite were either detected or not detected in culture supernatants during analysis, respectively.
