# Supplementary material for: In vivo functional analysis of a class A β-lactamase-related protein essential for clavulanic acid biosynthesis in Streptomyces clavuligerus
Source: PLoS One. 2019 Apr 23;14(4):e0215960. doi: 10.1371/journal.pone.0215960 (PMC6478378; doi:10.1371/journal.pone.0215960)
Supplement: S5 Table — (PDF) [file pone.0215960.s011.pdf]

S5 Table. LC-MS analysis of soy medium culture supernatants from wt *S. clavuligerus* and *Δcpe-INF* mutant strains for detecting 5*S* clavam production.

**Strain (plasmid)**[a]	**Metabolite**[b,c]		
	**2-Hydroxymethyl clavam** (m/z=144/212)	**Clavam- 2-carboxylate** (m/z=158/226)	**Clavulanic acid** (m/z=156/224)
**wt**	+	+	+
Δcpe-INF	+	+	-
***Δcpe-INF* (pSET:*cpe*[Sc])**	+	+	+
***Δcpe-INF* (pHM:*cpe*[Ct])**	+	+	-

[a] Select strains were fermented for 96 hours and culture supernatants were subjected to LC-MS analysis after imidazole derivatization.

[b] Metabolites and respective *m/z* values of corresponding imidazole derivatives/fragmentation products/ions are indicated, where (+ and -) indicate that ions corresponding to the metabolite were either present or was not detected in culture supernatants, respectively.

[c] The presence of 2-hydroxymethyl clavam and clavam-2-carboxylate was used to monitor 5*S* clavam production since the metabolites produced as part of a common biosynthetic pathway in *S. clavuligerus*.
